# Proactive personality and career crafting in Chinese medical professionals: a conditional process model of occupational stigma and organizational career management

**DOI:** 10.3389/fpsyg.2026.1876629

**Published:** 2026-08-11

**Authors:** Yue Yuan, Zemei Zhang, Nan Luo, Xuan Yu, Jing Huang

**Affiliations:** 1 College of State Governance, Southwest University, Chongqing, China; 2 School of Management, Xihua University, Chengdu, China; 3 Research Institute of International Economics and Management, Xihua University, Chengdu, China; 4 Business School, Sichuan University, Chengdu, China; 5 School of Economics and Management, Southwest Petroleum University, Chengdu, China

**Keywords:** career crafting, career exploration and decision-making self-efficacy, occupational stigma, organizational career management, proactive personality

## Abstract

**Background:**

Against the backdrop of healthcare reforms in China, medical professionals are increasingly required to manage their careers proactively. Proactive personality is associated with proactive career behaviors, while occupational stigma, a prevalent stressor, may shape career perceptions and choices. Despite its potential relevance, it remains unclear whether organizational career management may serve as a supportive contextual factor in such circumstances. This cross-sectional exploratory study examines the mediating role of career exploration and decision-making self-efficacy (CEDSE) in the relationship between proactive personality and career crafting, as well as the joint conditional associations of occupational stigma and organizational career management with this mediating pathway, drawing on Social Cognitive Career Theory and Cognitive Appraisal Theory of Stress.

**Methods:**

A convenience sample of 308 medical professionals working at county-level and grassroots healthcare institutions in Southwest China was recruited. Survey data were analyzed via SPSS 24.0, AMOS 22.0, and the PROCESS macro.

**Results:**

Proactive personality was positively associated with career crafting. CEDSE mediated the association between proactive personality and career crafting. Although the two-way interaction between occupational stigma and CEDSE reached marginal significance, the positive relationship between CEDSE and career crafting was more pronounced under higher levels of occupational stigma. Further three-way interaction analysis indicated that organizational career management was associated with a stronger conditional pattern. The overall index of moderated moderated mediation was statistically significant, suggesting that the indirect association of proactive personality with career crafting via CEDSE varies jointly with occupational stigma and organizational career management.

**Conclusion:**

This study provides preliminary cross-sectional evidence for the association between proactive personality and career crafting mediated by CEDSE. The indirect association tends to be stronger under high occupational stigma, especially when accompanied by sufficient organizational career management support. Building on two complementary theoretical perspectives of individual agency and organizational context, this framework offers tentative theoretical insights and research references for understanding career adaptation among medical professionals. It also provides exploratory implications for healthcare institutions seeking to build supportive working environments and address occupational stigma.

## Introduction

In the evolving landscape of Chinese healthcare, medical professionals navigate a paradoxical reality where unprecedented policy-driven opportunities intersect with intensifying professional challenges, making proactive career self-management increasingly necessary. The government’s 2023 healthcare reform initiative, particularly through its promotion of “Internet Plus Healthcare” and multi-location practice (General Office of the Central Committee of the Communist Party of China, 2023), has substantially expanded service delivery models and career pathways, granting practitioners unprecedented autonomy while simultaneously demanding greater personal responsibility for career development. However, this expanding professional autonomy coexists with the deepening crisis of occupational stigma, which has evolved alongside healthcare marketization: a profession once held in universal esteem now faces widespread public criticism, devaluation, and even targeted patient violence (Fang and Cao, 2020; Wang et al., 2020). Within this context of simultaneous opportunity and threat, Chinese medical professionals face mounting pressure to engage in self-directed career management and crafting behaviors to navigate uncertain professional trajectories effectively.

Based on this context, career crafting, defined as self-initiated, proactive behaviors that individuals undertake to shape their work experiences, tasks, and career paths (Akkermans and Hirschi, 2023; Chifor and Oprea, 2023), has emerged as a potentially important adaptive strategy for medical professionals. This raises a pivotal theoretical and practical question: what factors are associated with such proactive career behaviors? Existing research on the antecedents of career crafting remains relatively scarce and has primarily focused on proximal motivational or state-like factors, such as work commitment (Alarifi et al., 2024), career adaptability resources (Nalis et al., 2022), family motivation (Ge et al., 2025), and career calling (Ni et al., 2024). While these variables are indeed associated with career crafting under certain conditions, they do not address the more fundamental dispositional source of proactive tendencies. Accordingly, scholars have repeatedly called for examining distal personality traits like proactive personality as a broader, cross-situational antecedent of career self-management behaviors (Ge et al., 2023; Bateman and Crant, 1993).

To examine how dispositional traits relate to concrete action, we integrate two complementary theoretical perspectives that address the dual roles of personal agency and contextual influence. First, from a personal agency perspective, the Social Cognitive Career Theory, specifically its career self-management model (Lent and Brown, 2013; Lent et al., 1994), posits that distal personal inputs such as proactive personality are associated with career-adaptive behaviors through cognitive self-regulatory resources. The core mechanism in this process is career exploration and decision-making self-efficacy (CEDSE), which represents an individual’s confidence in successfully performing career management tasks, including career exploration, goal setting, and decision-making (Lent and Brown, 2013; Lent et al., 2016). Proactive personality is positively associated with self-efficacy because proactive individuals are more likely to seek out and accumulate relevant experiences, develop task-related skills, and build confidence in their abilities (Bandura, 1997), a relationship that has been consistently documented in academic and career decision-making contexts (Lin et al., 2014; Kim and Park, 2017). In turn, high CEDSE is linked to greater optimism toward change (Tims et al., 2014) and increased engagement in proactive career development activities (Rogers and Creed, 2011). We thus propose that CEDSE mediates the relationship between proactive personality and career crafting.

Second, we incorporate the contextual stress perspective to complement the agentic view, responding to persistent calls to recognize the equal importance of environmental factors in shaping career behavior (De Vos et al., 2019). Grounded in the Cognitive Appraisal Theory of Stress (Lazarus and Folkman, 1984; Lazarus, 2006), we examine how contextual factors may condition the relationship between self-efficacy and action. Occupational stigma, intensified by healthcare marketization and manifested through public criticism and patient violence (Fang and Cao, 2020; Wang et al., 2020), is fundamentally a harmful social phenomenon that inflicts widespread damage on individuals’ mental health and well-being (Wang et al., 2020; Sun et al., 2021). However, occupational stigma presents dual potential outcomes. On one hand, it triggers identity threat, psychological distress, and behavioral withdrawal (Wang et al., 2020; Miller and Kaiser, 2001). On the other hand, for individuals with high self-efficacy, it may be linked to identity repair and proactive career adjustment as a coping response. While the adverse nature of stigma itself is universal, its influence on the relationship between self-efficacy and proactive behavior varies, depending critically on the individual’s cognitive appraisal. We suggest that the pressure brought by occupational stigma may correlate with stronger career crafting tendencies among individuals with high CEDSE. According to cognitive appraisal theory of stress, when a stressor is appraised as a threat to one’s core professional identity, it may relate to a tendency to protect the self-concept (Lazarus and Folkman, 1984). In the specific context of healthcare, occupational stigma is primarily appraised as such an identity threat (Miller and Kaiser, 2001). This appraisal may be associated with defensive coping responses, where individuals draw on self-efficacy resources to reassert control and protect their professional self-concept, leading to a stronger positive linkage between CEDSE and career crafting. Conversely, for individuals with low CEDSE, stigma may exceed their coping capacity and correspond to a weaker association between the two variables. This leads to our first moderation hypothesis: occupational stigma may positively moderate the relationship between CEDSE and career crafting, such that the linkage becomes stronger under higher levels of occupational stigma.

Concurrently, the secondary appraisal process in cognitive appraisal theory of stress highlights the role of available coping resources after individuals perceive contextual threats. Organizational career management, understood as structured organizational support for career development (Sturges et al., 2002), serves as a critical contextual resource in this appraisal stage (Lazarus and Folkman, 1984; Kaiser et al., 2004), especially for professionals in organization-dependent careers (Noordegraaf, 2007; van Leeuwen, 2023). Aligned with the core connotation of secondary appraisal, it provides tangible resources (e.g., mentoring, clear career pathways) and perceived organizational support (Yildiz et al., 2024) that may help individuals more effectively translate coping motivation into concrete career actions. We therefore hypothesize that organizational career management moderates the moderating effect of occupational stigma on the relationship between CEDSE and career crafting, such that the positive moderating trend of occupational stigma is stronger when organizational career management is high rather than low.

By integrating these dual pathways outlined above, we propose a moderated moderated mediation model. This model posits that the indirect association of proactive personality with career crafting through CEDSE, the core agentic mechanism in social cognitive career theory (Lent and Brown, 2013), is conditioned by the interactive influence of occupational stigma and organizational career management. Grounded in cognitive appraisal theory of stress (Lazarus and Folkman, 1984), occupational stigma is appraised as an identity threat that may relate to individuals’ use of self-efficacy resources for career actions. Organizational career management, as a critical coping resource, is associated with the degree to which such tendencies translate into observable behaviors. Consistent with Bandura’s triadic reciprocal determinism (Bandura, 1978), we theorize that CEDSE as a personal resource shows the strongest linkage with proactive career behavior when high contextual pressure (i.e., occupational stigma) coincides with high contextual support (i.e., organizational career management). Thus, cognitive appraisal theory of stress complements social cognitive career theory by indicating when the core agentic process tends to have the strongest association with behavior, integrating personal agency and situational appraisal within a unified theoretical framework.

To summarize, this study examines an integrated moderated moderated mediation model that investigates the relationship between proactive personality and career crafting among medical professionals from county-level and grassroots healthcare institutions in Southwest China. Drawing on the self-management perspective of social cognitive career theory, we examine the mediating role of CEDSE between proactive personality and career crafting. Simultaneously, informed by cognitive appraisal theory of stress, we theorize that occupational stigma moderates the relationship between CEDSE and career crafting, and that this moderating effect is further moderated by organizational career management. Moreover, this framework describes how the indirect association of proactive personality with career crafting via CEDSE is systematically shaped by the interplay of occupational stigma and organizational career management, thereby integrating personal agency with contextual appraisal. The study aims to contribute to career theory by providing preliminary cross-sectional evidence for a context-sensitive self-regulatory process, while offering exploratory directions that, pending future longitudinal and experimental research, may eventually inform relevant academic research on career crafting in settings marked by occupational stigma.

### Proactive personality and career crafting

Proactive personality reflects an individual’s behavioral tendency to actively seek opportunities and take action. Individuals with this personality usually maintain a proactive action orientation, seeking to break through the constraints of the external environment to achieve environmental and self-improvement (Bateman and Crant, 1993). As such, proactive personality represents an instrumental trait aimed at mastering the environment and has been positively linked to multiple forms of proactive behavior. Career crafting is precisely the proactive behavior of self-managing one’s career (Tims and Akkermans, 2020) that individuals adopt to promote career sustainability amid rapidly changing work environments (van Leeuwen et al., 2020; Parker et al., 2017). Specifically, career crafting is defined as a set of proactive behaviors that create or extend task, relational, and cognitive resources to align one’s career with personal interests, strengths, values and needs. It encompasses four dimensions: changing relational boundaries, utilizing relational resources, reflecting on positive career meaning, and extending task boundaries (Lee et al., 2021).

Specifically, proactive personality is positively associated with the behavioral manifestations of career crafting. First, proactive personality relates to individuals’ tendency to adjust relational boundaries and utilize relational resources. Proactive individuals tend to expand their social networks to obtain more favorable job outcomes (Le and Lin, 2023). As a kind of embedded social resource (Lin, 2002), interpersonal networks determine the range of accessible information and individual environmental influence (Brass, 2001; Coleman, 1988). Second, proactive personality correlates with individuals’ reflection on positive career meaning. People with high proactive personality tend to select, adjust and redefine work to reshape their surroundings (Bakker et al., 2012; Parker and Ohly, 2008), and devote themselves to endowing work with deeper meaning (Bateman and Crant, 1993; Crant, 2000). Prior studies have confirmed a positive correlation between proactive personality and meaningful work (Hakimi, 2020; Vermooten et al., 2019). Finally, extending task boundaries means career crafters voluntarily take on extra duties and assume new career-related responsibilities within the organization (Lee et al., 2021). Proactive individuals are also inclined to expand their role breadth and take more work responsibilities (Parker and Sprigg, 1999). For medical professionals, this may involve undertaking additional work in education, administration or research besides routine clinical tasks (Wass, 2006). Meta-analytic results have shown that proactive personality is positively correlated with extra-role behaviors such as voice, taking charge and creative performance (Spitzmuller et al., 2015). Accordingly, it is plausible that medical professionals with higher proactive personality are more likely to engage in task boundary expansion. We thus propose the following hypothesis:

*H1*: Proactive personality is positively associated with career crafting.

### The mediating role of career exploration and decision-making self-efficacy

Career exploration and decision-making self-efficacy (CEDSE) refers to individuals’ perceived capability to complete relevant procedures for matching jobs with personal abilities, interests and values (Lent and Brown, 2013). It contains two sub-dimensions: decisional self-efficacy and decisional coping efficacy (Lent et al., 2017). Decisional self-efficacy is defined as confidence in career selection, adaptation and adjustment, including self-efficacy for career decision-making (Betz et al., 1996), job searching (Saks et al., 2015) and multi-role management (Cinamon, 2006). Decisional coping efficacy reflects beliefs in one’s ability to handle difficulties after making career choices. Lent and Brown developed the career self-management model based on Social Cognitive Career Theory (Lent and Brown, 2013; Lent et al., 1994), which explains the developmental mechanisms of individual careers, behaviors and choices.

Social cognitive career theory holds that individuals’ perceived capability to perform certain behaviors correlates with their actual engagement in those behaviors (Lent et al., 1994). Within the career self-management model, CEDSE acts as a core factor linking personal interests, goals and real actions. As distal dispositional factors, personality traits are linked to career behaviors via CEDSE. From the perspective of individual initiative, self-efficacy has been conceptualized as a key source of “can-do” motivation (Parker et al., 2010) and serves as a vital intermediate linkage between personal behavioral tendencies and concrete actions (Frese and Fay, 2001). Individuals with strong proactive personality tend to have greater confidence in career exploration and decision-making, and actively solve career-related problems on their own initiative (Crant, 2000; Park, 2015). Existing evidence has verified the positive correlation between proactive personality and career decision-making self-efficacy, as well as general career self-efficacy (Kim and Park, 2017; Acar, 2022). In addition, proactive engagement is accompanied by potential interpersonal challenges such as others’ resistance and doubt when adjusting work modes (Frese et al., 2007). Nevertheless, self-efficacy is correlated with stronger persistence and willingness to overcome difficulties (Bandura, 1997).

As a form of proactive career self-management, career crafting also involves various obstacles during task expansion and relational adjustment. With higher CEDSE, individuals tend to engage in forethought, intentional action, self-reflection and self-regulation. These capabilities are associated with more active participation in career selection and development (Bandura, 2001; Bandura, 2006). CEDSE is also correlated with a solid basis for career exploration and planning, enabling individuals to anticipate future development, reflect on the fit between self and environment, and set and revise personal goals (Bandura, 2001; Bandura, 2006). Prior research has demonstrated that domain-specific self-efficacy correlates with corresponding proactive actions. For instance, job-search self-efficacy links to proactive job seeking (Kanfer et al., 2001), and role-breadth self-efficacy correlates with improvement suggestions and proactive problem-solving (Axtell et al., 2000; Parker and Liao, 2016). Therefore, we propose that CEDSE may serve as a critical motivational mechanism linking proactive personality and career crafting, and propose the following hypothesis:

*H2*: Career exploration and decision-making self-efficacy (CEDSE) mediates the relationship between proactive personality and career crafting.

### The moderating role of occupational stigma and organizational career management

Like all proactive behaviors, career crafting is shaped by a combination of personal and situational forces (e.g., Griffin et al., 2010; McAllister et al., 2007). While proactive personality represents a trait in which individuals are relatively unconstrained by situational forces (Bateman and Crant, 1993), proactive behaviors are influenced not only by a person’s personality but also by a person’s motivation in a given context (Bindl and Parker, 2011). The model of career self-management based on social cognitive career theory suggests that obstacles or supportive factors may be associated with the process of linking proximal motivation to career exploration or decision-making behaviors in context (Lent et al., 1994; Lent et al., 2016). Occupational stigma is a context that is closely related to and salient for Chinese medical professionals (Sun et al., 2021). At the same time, with diverse career preferences and challenging external environments, medical professionals are increasingly dependent on organizational guidance and support (Noordegraaf, 2011). In the following discussion, we discuss the potential roles of occupational stigma and organizational career management in relation to career crafting in more detail.

### The moderating role of occupational stigma

As a discrediting attribute or mark, stigma is a derogatory social evaluation (Goffman, 1963; Kreiner et al., 2022). Traditionally, occupational stigma has been associated with dirty work, which society views as repulsive, distasteful, or degrading (Kreiner et al., 2006; Ashforth and Kreiner, 1999). However, even seemingly innocuous occupations may carry some element of stigmatization (Hughes, 1970; Meara, 1974); once practitioners’ behavior goes against established societal norms and values, they may carry a moral stigma (Ashforth, 2018; Link and Phelan, 2001). Chinese medical professionals have experienced a stigmatization process from “white angels” to “white wolves” (Wang et al., 2020). After the market-oriented reform of China’s healthcare system, medical professionals were accused of widespread misconduct and deviation from important social norms, thus triggering a process of stigmatization (Wang et al., 2020). Current research suggests that conceptualizing stigma as a stressor can help understand and cope with stigma (Miller and Kaiser, 2001; Kaiser et al., 2004). Importantly, occupational stigma is fundamentally a harmful social phenomenon that has been consistently associated with negative outcomes including mental distress, burnout, and turnover intention among medical professionals (Wang et al., 2020; Sun et al., 2021). While the harm of stigma itself is universal, individuals’ responses to stigma are not monolithic; they depend critically on cognitive appraisal and available coping resources. Occupational stigma thus has a dual possibility. On one hand, its harmful effects on mental health, burnout, and turnover are well established (Wang et al., 2020; Miller and Kaiser, 2001). On the other hand, for individuals with high CEDSE, the pressure associated with stigma may also be linked to identity repair and proactive career adjustment as a coping response.

The cognitive appraisal theory of stress posits that individuals identify whether an event is potentially threatening through primary appraisal (Lazarus and Folkman, 1984; Lazarus, 2006). When medical professionals recognize occupational stigma as a threat to their core professional identity (Goffman, 1963; Dovidio et al., 2000), this recognition may be associated with their engagement in identity work (Petriglieri, 2011). In the workplace and institutional settings, identity work involves a range of strategic behaviors that individuals adopt to maintain the integrity of their self-concept (Caza et al., 2018). For individuals with high CEDSE, those who are confident in their ability to manage their careers, this identity threat may be associated with proactive identity repair strategies. They may tend to reevaluate and reinterpret the meaning of their work, rethink their career trajectories, and engage in career crafting to reassert control over their professional identities (Morales and Lambert, 2013; Toyoki and Brown, 2013). In contrast, for individuals with low CEDSE, occupational stigma may be associated with diminished coping capacity and passive withdrawal rather than proactive adaptation. Thus, the positive relationship between CEDSE and career crafting may be stronger under high occupational stigma. Conversely, when occupational stigma is at a low level, medical professionals’ motivation to protect their identity from threats may be diminished, and they may feel less need to take adaptive career actions (Lent and Brown, 2013); consequently, the positive association between CEDSE and career crafting may be weaker. Thus, the following hypothesis is put forth:

*H3a*: Occupational stigma positively moderates the relationship between CEDSE and career crafting.

### The moderating role of organizational career management

Organizational career management refers to organizational arrangements designed to support employees’ career development (Arnold, 1997), encompassing a range of formal activities such as training courses, assessment centers, mentoring, and career counseling (Sturges et al., 2002). In contrast, career self-management consists of individual-driven activities including information gathering, career problem-solving, and decision-making (Kossek et al., 1998). It covers two major types of behaviors: optimizing current work and preparing for job mobility. In this sense, career crafting can be regarded as a typical form of active career self-management, as it involves both present work and long-term career development (De Vos et al., 2019).

It should be noted that organizational career management and career self-management are not mutually exclusive; in some cases, organizational career management may be positively associated with career self-management behaviors (Sturges et al., 2002). Although the trend toward individual agency has brought the organization somewhat to the background, the role of organizations in individual career development should not be underestimated (De Vos et al., 2019), especially in China, where most medical professionals are affiliated with formal healthcare institutions (National Health Commission of the People's Republic of China, 2024). According to cognitive appraisal theory of stress, the cognitive appraisal process imbues meaning on potentially stressful events and is associated with one’s path of action (or inaction) in dealing with these events, and primary and secondary appraisals are interdependent in this process (Lazarus and Folkman, 1984; Lazarus, 2006). When medical professionals appraise occupational stigma as an identity threat in the primary appraisal stage, they may simultaneously evaluate available coping resources in the secondary appraisal stage.

Crucially, organizational career management does not merely buffer the negative effects of stigma; rather, it provides tangible and social resources that may help individuals translate identity protection motivation into concrete proactive action. Research suggests that organizational career management may be associated with the accumulation of internal resources, including knowledge, attitudes, skills, experience, and networks (Yildiz et al., 2024; Bagdadli and Gianecchini, 2019). When medical professionals receive organizational career management support, they are more likely to believe they have sufficient resources to cope with the threats posed by occupational stigma and, therefore, may be more likely to perceive occupational stigma as a manageable challenge, which may be associated with more proactive career self-management behaviors.

Thus, the positive moderating effect of occupational stigma on the relationship between CEDSE and career crafting tends to be stronger when organizational career management is high. Sturges et al. (2002) noted that employees who receive career management support from their employers are more likely to engage in career management behaviors aimed at facilitating their careers within their organizations. Conversely, when organizational career management is limited, medical professionals may perceive insufficient organizational support and may be less likely to adopt proactive career self-management model behaviors when facing greater risk and stress (Canboy et al., 2023); accordingly, the positive moderating effect of occupational stigma between CEDSE and career crafting relationship tends to be weaker. In summary, we propose the following hypothesis:

*H3b*: Organizational career management positively moderates the moderating role of occupational stigma between CEDSE and career crafting. Specifically, high levels of organizational career management will strengthen the positive moderating effect of occupational stigma.

### The integrated moderated mediation effect

Combined with all the above hypotheses, this study examines an integrated moderated mediation model that specifies the conditional nature of the core mediating pathway. Drawing on Social Cognitive Career Theory (Lent et al., 1994) and cognitive appraisal theory of stress (Lazarus and Folkman, 1984), we posit that the indirect effect of proactive personality on career crafting via CEDSE may reflect a self-regulatory process (Lent and Brown, 2013). This process is contextualized by a primary appraisal of threat, operationalized as occupational stigma, and a secondary appraisal of coping resources, operationalized as organizational career management.

Theoretically, this conditional indirect effect is expected to be strongest under the specific circumstance where a high primary appraisal of threat coincides with a high secondary appraisal of organizational support. Under this joint condition, individuals may be simultaneously driven by identity threat to initiate proactive adaptation and enabled by sufficient organizational resources to engage in career crafting (Sturges et al., 2002; Petriglieri, 2011). Thus, the integrated model describes a contextually embedded self-regulatory process in which personal agency and organizational resources may interact in relation to the realization of career crafting.

We therefore put forward the final hypothesis:

*H3c*: The indirect effect of proactive personality on career crafting via CEDSE is conditionally moderated by the interactive effect of occupational stigma and organizational career management. Specifically, the indirect effect is strongest when both occupational stigma and organizational career management are at high levels.

**Figure 1:**
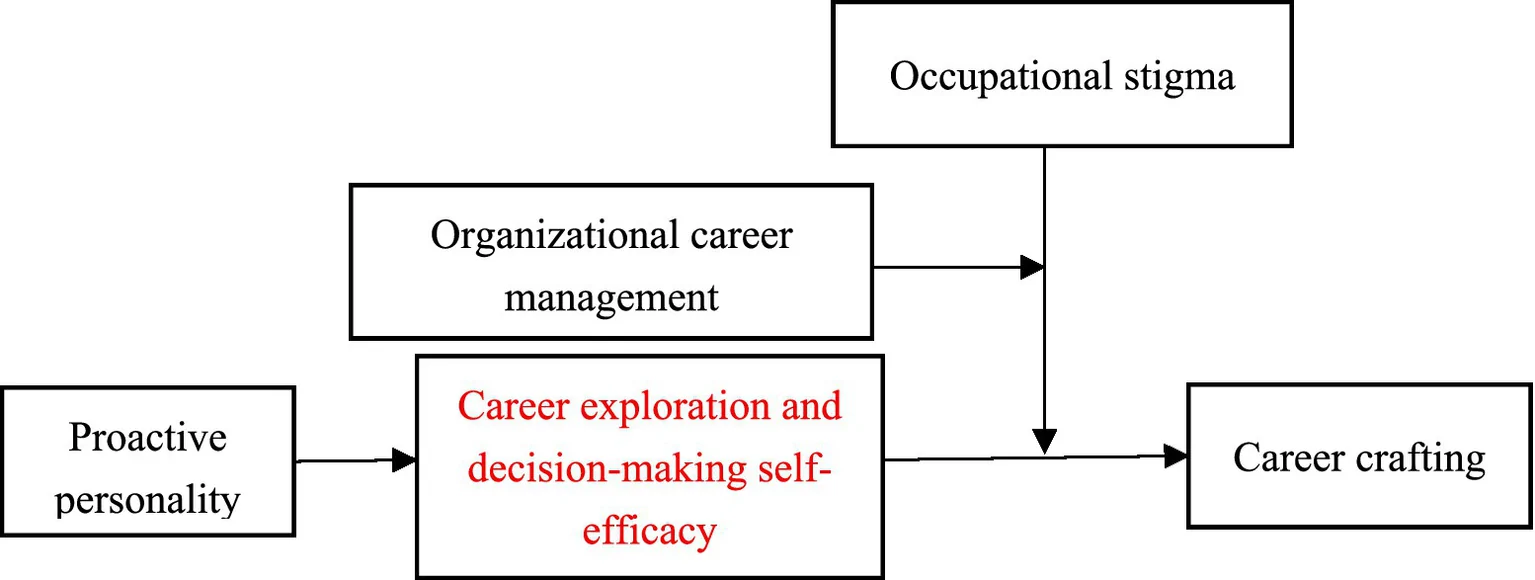
The proposed model.

The conceptual model of the study is presented in Figure 1.

## Methodology

### Participants and data collection

This exploratory cross-sectional study employed a multistage sampling strategy combining organization-level random selection and participant-level convenience sampling. First, twelve healthcare service organizations were randomly selected from forty healthcare service organizations in a county-level city in Southwest China. The selected institutions included both public and private hospitals spanning six hospital grades (Level 3A through Level 1B), which provided substantial variation in healthcare organizational characteristics. Within each selected organization, convenience sampling was used to recruit participants. Eligible participants were healthcare professionals who were formally employed in the participating institutions and voluntarily agreed to participate in the study. No minimum tenure requirement was imposed, as the focus was on capturing the full range of medical professionals in this county-level region.

Data collection was carried out from June 2024 to September 2024. Paper questionnaires were distributed to eligible healthcare professionals through hospital administrators across departments within each institution. Participation was entirely voluntary, and no incentives were provided. To minimize concerns regarding disclosure and enhance the authenticity and completeness of responses, informed consent, anonymity, and confidentiality were emphasized throughout the survey process. The questionnaire measured the core study variables (proactive personality; CEDSE; occupational stigma; organizational career management; and career crafting) as well as demographic information and other control variables. Given the cross-sectional design and reliance on self-report data, we took several steps to mitigate potential common method bias: the order of scale items was randomized to disrupt possible response patterns and reduce method-specific covariance. A total of 320 paper questionnaires were distributed. After excluding questionnaires with substantial missing data, inconsistent responses, or invalid completion patterns, 308 valid questionnaires were retained, yielding an effective response rate of 96.25% (308/320).

The detailed demographic characteristics of the sample are presented in Supplementary Table S1. The sample comprised 65 males (21.1%) and 243 females (78.9%). This gender distribution is broadly consistent with the demographic profile of China’s healthcare workforce: according to the 2023 China Health Statistics Yearbook, women constitute 73.2% of all health technicians nationally (National Health Commission of the People's Republic of China, 2023). This female predominance is particularly evident in the nursing profession, which made up 33.4% of our sample and is overwhelmingly female nationwide. The age distribution ranged from 18 to 64 years, with the largest group being 25–30 years (32.8%), followed by 20–25 years (17.9%) and 30–35 years (14.6%). Regarding education level, the majority held a bachelor’s degree (51.6%) or junior college degree and below (45.5%), with only a small proportion holding a master’s degree (1.6%) or doctoral degree (1.3%).

In terms of job type, nurses constituted the largest group (33.4%), followed by physicians (27.9%) and other professional and technical personnel (18.8%). The majority of participants held junior professional titles (71.4%), with 19.8% at the intermediate level, 7.5% at the associate senior level, and 1.3% at the senior level. Most participants were frontline general staff (69.8%), with smaller proportions in middle management (18.2%), junior management (7.5%), and senior management (4.5%). Both occupational tenure and organizational tenure were widely distributed across all experience brackets, with roughly 60% of participants having 10 years or less of professional experience.

Regarding organizational characteristics, 160 participants (51.9%) worked in public hospitals and 148 (48.1%) in private hospitals. In terms of hospital level, the majority were from primary hospitals (81.5% total; 58.1% Level 1A, 23.4% Level 1B), with smaller proportions from secondary hospitals (11.7% combined for Level 2A and 2B) and tertiary hospitals (6.8% combined for Level 3A and 3B). This distribution aligns with the typical healthcare institutional structure in county-level regions of Southwest China, where primary-level hospitals serve as the main healthcare providers.

Notably, medical professionals working in county-level and grassroots healthcare institutions constitute a substantial and policy-relevant segment of China’s healthcare workforce. According to the 2024 China Health Statistics Bulletin, grassroots healthcare institutions (township hospitals, community health centers/stations, and village clinics) employ approximately 3.75 million health workers, accounting for 28.8% of China’s 13.02 million health technicians. When combined with staff from county-level secondary hospitals, this group represents over half of China’s total health workforce. Our sample, which includes primary, secondary, and tertiary hospitals in a county-level city, provides valuable insights into this understudied population.

### Measures

We used validated Western scales and adopted a rigorous multi-step cross-cultural adaptation procedure to ensure conceptual equivalence, linguistic accuracy, and contextual appropriateness in the Chinese medical context. Initially, two independent bilingual scholars with PhD degrees in management independently translated the scales from English to Chinese and then back-translated them to English, with any discrepancies in wording or meaning resolved through discussion and consensus, and additional input from a professor specializing in English language studies to ensure linguistic precision. Following the translation process, healthcare management scholars and frontline medical professionals, conducted a comprehensive content validity review of all translated scales, evaluating each item on whether it accurately measured the intended construct, whether the wording was clear and understandable to Chinese medical professionals, and whether it was culturally appropriate for the healthcare setting, and minor revisions were made to several items based on their feedback to improve clarity and contextual fit. The revised scales were then pre-tested on a small sample of 15 medical professionals to assess face validity and response clarity, with further minor adjustments made to enhance readability and align with Chinese reading habits, and confirmatory factor analyses were subsequently conducted to verify the factor structure of all scales as detailed in the subsequent sections.

Proactive personality was measured using a 10-item scale developed by Seibert et al. (1999). Responses were recorded on a 7-point Likert scale ranging from 1 (strongly disagree) to 7 (strongly agree). A sample item is: “I am constantly on the lookout for new ways to improve my life.” The scale demonstrated excellent internal consistency in this study (Cronbach’s *α* = 0.905, composite reliability (CR) = 0.911).

CEDSE was measured using a 12-item scale developed by Lent et al. (2016), which includes two dimensions: decisional self-efficacy and decisional coping efficacy. Sample items include: “I have confidence in my ability to figure out which career options could provide a good fit for my personality” (decisional self-efficacy) and “I have confidence in my ability to cope with the disappointment if my first choice does not work out” (decisional coping efficacy). Participants responded on a 10-point Likert scale ranging from 1 (strongly disagree) to 10 (strongly agree). Confirmatory factor analysis (CFA) was conducted to compare alternative measurement models for CEDSE. A one-factor model demonstrated acceptable fit (χ^2^/df = 3.214, CFI = 0.972, TLI = 0.965, RMSEA = 0.085, SRMR = 0.028), and the two sub-dimensions were highly correlated (r = 0.861). Consistent with the original scale development study (Lent et al., 2016), coping efficacy items showed high cross-loadings on the decisional self-efficacy factor (0.61–0.74) and did not contribute unique variance beyond the general factor. Therefore, we used the total CEDSE score in subsequent analyses. The scale exhibited excellent internal consistency (Cronbach’s *α* = 0.974, CR = 0.973).

Occupational stigma was measured using six items adapted from Shantz and Booth (2014), with the reference population changed from “call center employees” to “medical professionals” to fit the study context. A sample item is: “Most people who are not medical professionals have a lot more negative thoughts about medical professionals than they actually express.” Responses were recorded on a 7-point Likert scale ranging from 1 (strongly disagree) to 7 (strongly agree). The scale demonstrated good internal consistency (Cronbach’s *α* = 0.882, CR = 0.879).

Organizational career management was measured using a 10-item scale developed by Sturges et al. (2002). Responses were recorded on a 5-point Likert scale ranging from 1 (strongly disagree) to 5 (strongly agree). A sample item is: “I have been given a personal development plan.” The scale exhibited excellent internal consistency (Cronbach’s α = 0.943, CR = 0.942).

Career crafting was measured using a 15-item scale developed by Lee et al. (2021), which includes four dimensions: changing relational boundaries, utilizing relational resources, reflecting positive career meaning, and expanding task boundaries. Sample items include: “I make an effort to get to know people whose career I admire” (changing relational boundaries), “I seek professional coaching from those whose careers I admire” (utilizing relational resources), “I think about the ways in which my career positively impacts my life” (reflecting positive career meaning), and “I choose to take on additional tasks at work” (expanding task boundaries). Participants responded on a 7-point Likert scale ranging from 1 (strongly disagree) to 7 (strongly agree). CFA was conducted to compare three alternative measurement models for career crafting: a one-factor model, a four-factor correlated model, and a higher-order model (four first-order factors loading onto a single second-order factor). The higher-order model demonstrated the best fit (χ^2^/df = 3.165, CFI = 0.966, TLI = 0.954, RMSEA = 0.084, SRMR = 0.033), consistent with the original validation study (Lee et al., 2021). The four dimensions were highly correlated, so we used the total score of the higher-order factor in subsequent analyses. The scale exhibited excellent internal consistency (Cronbach’s *α* = 0.965, CR = 0.974).

Consistent with previous research on career crafting (Alarifi et al., 2024; Ni et al., 2024; Ge et al., 2023), we controlled for demographic and job-related variables including gender, age, education level, job type, title level, job hierarchy, occupational tenure, and organizational tenure. Given the Chinese healthcare context, we also controlled for hospital nature (public vs. private) and hospital level. All nominal categorical variables were dummy-coded for regression analyses, with “Male,” “Physician,” “Public,” and “Level 3A” as the reference groups. Ordinal categorical variables (age, education level, title level, job hierarchy, occupational tenure, organizational tenure) were treated as continuous.

### Statistical analyses

We employed a comprehensive analytical strategy to test our hypotheses, which included descriptive statistics, measurement model validation, correlation analysis, nested data structure evaluation, hypothesis testing, robustness checks, and alternative model comparisons. All analyses were conducted using SPSS 24.0 and AMOS 22.0, and all statistical tests were two-tailed with a significance level set at *p* < 0.05.

First, descriptive statistics were calculated to summarize the sample characteristics and the distributions of all study variables. Frequencies and percentages were reported for categorical variables, while means and standard deviations (SD) were reported for continuous variables. Internal consistency reliability was assessed using Cronbach’s alpha coefficients and composite reliability (CR) for all scales. Next, confirmatory factor analysis (CFA) in AMOS was conducted to validate the measurement model and assess convergent and discriminant validity, and Pearson correlation analysis was then performed to examine the bivariate relationships among all study variables and control variables. Given that participants were clustered within 12 healthcare institutions, we systematically evaluated the nested data structure. The intraclass correlation coefficient (ICC(1)) for the outcome variable (career crafting) was calculated to assess between-institution variance, and a one-way analysis of variance (ANOVA) was used to test for significant differences in career crafting scores across hospitals. To further confirm the robustness of our results, we also conducted a linear mixed model with hospital ID specified as a random intercept, controlling for all study variables and covariates.

Subsequently, we tested our hypothesized moderated mediation model using Hayes’s PROCESS macro for SPSS. All continuous predictor variables involved in moderation analyses were mean-centered prior to constructing interaction terms to mitigate multicollinearity and facilitate interpretation. Specifically, the direct effect of proactive personality on career crafting and the mediating role of CEDSE were tested using PROCESS Model 4, the moderating effect of occupational stigma on the CEDSE-career crafting relationship was tested using PROCESS Model 1, and the three-way interaction effect (CEDSE × occupational stigma × organizational career management) and the moderated moderated mediation model were tested using PROCESS Model 18. All mediation, moderation, and conditional indirect effect analyses used 10,000 bias-corrected bootstrap resamples to generate 95% confidence intervals, and effect sizes were reported alongside confidence intervals to provide a more comprehensive assessment of the magnitude of the relationships.

To further ensure the sensitivity and reliability of our findings, we conducted a comprehensive set of robustness checks. We compared models with and without control variables, as well as stepwise regression models, to confirm the stability of the main effects and interaction effects, and calculated variance inflation factor (VIF) and tolerance values to assess potential multicollinearity issues. We also used studentized residuals and Cook’s distance to identify and evaluate the impact of potential outliers, and re-estimated the full model using HC2 heteroscedasticity-consistent standard errors, which provide optimal Type I error control for samples of this size. Additionally, we tested three alternative models to verify the plausibility of our theoretical framework: a reverse-path model examining whether career crafting predicts proactive personality, which in turn predicts CEDSE; a competitive model specifying CEDSE as the antecedent and proactive personality as the moderator predicting career crafting; and a simpler baseline moderation model examining occupational stigma as a direct predictor of career crafting with organizational career management as a standard moderator.

Finally, we employed a two-stage analytic strategy throughout this study: first, CFA in AMOS to validate the measurement model; second, regression-based analysis using the PROCESS macro with composite scores for all variables. This approach is widely accepted in organizational psychology (Preacher and Hayes, 2008; Hayes, 2013). We chose this regression-based approach over latent-variable structural equation modeling (SEM) for three key reasons. First, our model involves product terms for two-way and three-way interactions. Estimating such interactions within an SEM framework requires complex product-indicator methods that often suffer from convergence problems and demand large sample sizes (Marsh et al., 2004). PROCESS, by contrast, constructs product terms directly from composite scores and uses robust ordinary least squares regression, providing stable estimates and bootstrap confidence intervals for conditional effects. Second, measurement error attenuates interaction effects toward zero according to classical test theory (Aiken and West, 1991; Cohen et al., 2003), so the significant interactions we observed are likely conservative estimates, and the true effect sizes may be larger. Third, our CFA demonstrated excellent model fit and high factor loadings, and all scales had high reliability (Cronbach’s *α* > 0.88), indicating that composite scores are psychometrically sound and the potential bias introduced by using them is minimal. The PROCESS macro also uses bias-corrected bootstrapping (Preacher and Hayes, 2008), which partially mitigates the impact of non-normality and measurement error. Given these considerations, robustness in handling interactions, transparency about attenuation effects, and strong psychometric support for our composite scores, we deem this approach methodologically rigorous and appropriate for testing our specific hypotheses.

### Ethics approval

This study received ethical approval from the Ethics Review Committee, Department of Psychology, Southwest University (No. H24216). All participants provided written informed consent after being informed about research purposes, voluntary participation, and data confidentiality measures. To ensure anonymity, questionnaires used coded identifiers instead of personal names, and raw data were stored on password-protected servers with access limited to the research team.

## Result

### Means, standard deviations, and correlations

Table 1 presents the descriptive statistics (means, standard deviations) and Pearson correlation coefficients for all study variables. As shown, proactive personality was significantly positively associated with CEDSE (r = 0.437, *p* < 0.001) and career crafting (r = 0.577, *p* < 0.001). Consistently, CEDSE was significantly positively associated with career crafting (r = 0.484, *p* < 0.01). All bivariate correlations were in the theoretically expected directions, providing preliminary support for our hypothesized relationships. Furthermore, we calculated latent variable correlations using maximum likelihood estimation in AMOS. To obtain more robust interval estimates, we employed bias-corrected bootstrapping with 5,000 resamples to generate 95% confidence intervals for the standardized latent correlations. The results are presented in Table 2. The pattern of latent correlations was consistent with the Pearson correlations, further confirming the preliminary associations among the study constructs.

**Table 1:** Descriptive statistics and correlations of study variables.

Variables	1	2	3	4	5
1. Proactive personality	(0.713)				
2. CEDSE	0.437^***^	(0.865)			
3. Career crafting	0.577^***^	0.484^**^	(0.846)		
4. Occupational stigma	0.302^***^	0.088	0.302^***^	(0.742)	
5. Organizational career management	0.486^***^	0.480^***^	0.525^***^	0.246^***^	(0.787)
*Mean*	4.687	7.481	5.118	4.581	3.538
*SD*	1.057	1.654	1.144	1.208	0.801

*N* = 308, ** *p* < 0.01, *** *p* < 0.001. The values in parentheses are the square roots of the average variance extracted (AVE).

**Table 2:** Inter-factor correlations and 95% confidence intervals.

Variables	1	2	3	4	5
1. Proactive personality	1				
2. CEDSE	0.445^***^ [0.302, 0.573]	1			
3. Career crafting	0.624^***^ [0.462, 0.735]	0.478^***^ [0.338, 0.596]	1		
4. Occupational stigma	0.343^***^ [0.189, 0.501]	0.106 [−0.044, 0.257]	0.317^***^ [0.167, 0.469]	1	
5. Organizational career management	0.542^***^ [0.414, 0.645]	0.500^***^ [0.361, 0.613]	0.541^***^ [0.378, 0.676]	0.299^***^ [0.150, 0.435]	1

*N* = 308, ^***^ *p* < 0.001.

### Measurement model

We conducted confirmatory factor analysis (CFA) in AMOS to validate the measurement model. Consistent with the original scale validation studies (Lent et al., 2016; Lee et al., 2021), career crafting was specified as a higher-order latent variable with four first-order dimensions, while proactive personality, CEDSE, occupational stigma, and organizational career management were specified as first-order latent variables. We compared six alternative measurement models to assess discriminant validity (see Supplementary Table S2 for full results). The hypothesized five-factor model demonstrated acceptable fit to the data (χ^2^/df = 2.164, RMSEA = 0.062, SRMR = 0.056, CFI = 0.908, IFI = 0.909, TLI = 0.902) and showed substantially better fit than all more parsimonious models. These results indicate that the five constructs are empirically distinct.

We assessed the reliability and validity of the measurement scales. The scales for proactive personality, career exploration and decision-making self-efficacy (CEDSE), occupational stigma, organizational career management, and career crafting all demonstrated sound psychometric properties (see Supplementary Table S3 for full details). First, the scales showed good internal consistency reliability, with Cronbach’s alpha coefficients for all variables exceeding the recommended threshold of 0.70 (Hair et al., 2019). Second, convergent validity was established, as the average variance extracted (AVE) for each construct surpassed the benchmark of 0.50. The specific AVE values were 0.509 for proactive personality, 0.748 for CEDSE, 0.551 for occupational stigma, 0.619 for organizational career management, and 0.716 for career crafting (all AVE ≥ 0.509), supporting acceptable convergent validity across all constructs (Segars, 1997). Finally, discriminant validity was assessed by comparing the square root of each construct’s AVE (presented in parentheses on the diagonal in Table 2) with its correlations with all other constructs (the off-diagonal values). The results indicated that the square root of each AVE was greater than its corresponding inter-construct correlations, supporting good discriminant validity. As a stricter test of discriminant validity, we also examined the bias-corrected bootstrap 95% confidence intervals for the inter-construct correlations (Table 2). None of the confidence intervals included 1, providing additional support for discriminant validity (Anderson and Gerbing, 1988). In summary, the measurement model met acceptable standards for reliability, convergent validity, and discriminant validity.

### Common method bias

We implemented both procedural and statistical controls to address potential common method bias. Procedurally, we randomized the order of scale items during survey administration and emphasized anonymity and confidentiality to reduce response bias. Statistically, we employed a two-step approach to assess common method bias. First, Harman’s single-factor test was conducted. An unrotated principal component analysis revealed that the first factor explained only 38.73% of the total variance, which is below the recommended threshold of 40% (Podsakoff et al., 2003), providing preliminary evidence that common method bias was not a dominant concern. Second, we used the unmeasured latent method factor technique. We added a common method variance (CMV) factor to the baseline five-factor model, with all indicators loading on this additional latent factor. The six-factor model with the CMV factor yielded the following fit indices: χ^2^/df = 2.098, RMSEA = 0.060, CFI = 0.913, IFI = 0.914, TLI = 0.908. Compared with the five-factor baseline model (χ^2^/df = 2.164, RMSEA = 0.062, CFI = 0.908, IFI = 0.909, TLI = 0.902), the improvements in model fit were negligible (ΔCFI = 0.005, ΔIFI = 0.005, ΔTLI = 0.006, ΔRMSEA = −0.002), all well below the conventional threshold of 0.01 for a meaningful improvement. These consistent results indicate that common method bias does not significantly affect the interpretation of our findings.

### Nested data structure evaluation

Given that participants were clustered within 12 healthcare institutions, we evaluated the nested data structure prior to hypothesis testing. The intraclass correlation coefficient (ICC(1)) for the outcome variable (career crafting) was calculated as 0 (MSB = 0.828, MSW = 1.327), indicating that between-institution variance was negligible. A one-way analysis of variance (ANOVA) also confirmed that there were no significant differences in career crafting scores across the 12 healthcare institutions (*F* (11, 296) = 0.624, *p* = 0.808).

We further conducted a linear mixed model with hospital ID specified as a random intercept, controlling for all study variables and covariates. The three-way interaction effect (CEDSE × occupational stigma × organizational career management) remained significant in this model (B = 0.050, SE = 0.016, t = 3.113, *p* = 0.002), and the random intercept variance was estimated at 0.000 (noted as redundant by SPSS), indicating no residual between-institution variance after controlling for hospital nature and hospital level. These results collectively demonstrate that no substantial nesting effects exist in the data, and individual-level analysis is appropriate.

### Test of main relationship

To examine the associations among the study variables, a set of hierarchical regression analyses was conducted. All interaction terms were mean-centered prior to analysis to mitigate multicollinearity. The results are presented in Table 3. Model 1 included only proactive personality without control variables, and proactive personality was positively associated with career crafting (*β* = 0.577, *p* < 0.001). Model 2 further added CEDSE, occupational stigma, organizational career management and all interaction terms while excluding controls, and the three-way interaction reached statistical significance (*β* = 0.158, *p* < 0.01). Model 3 incorporated all demographic and occupational control variables alongside proactive personality. After accounting for covariates, proactive personality remained positively associated with career crafting (*β* = 0.601, *p* < 0.001). The model explained 38.5% of the variance in career crafting (R^2^ = 0.385). Compared with the control-only baseline model (R^2^ = 0.056), ΔR^2^ = 0.329 indicated that proactive personality explained additional variance in career crafting beyond demographic and occupational factors. Subsequent hierarchical models sequentially entered the mediator, moderators and interaction terms. Detailed results for these stepwise models are reported in the following moderation and moderated mediation sections.

**Table 3:** Hierarchical regression results predicting career crafting.

Predictor	M1	M2	M3	M4	M5	M6	M7
Control variables	No	No	Yes	Yes	Yes	Yes	Yes
PP	0.577^***^	0.316^***^	0.601^***^	0.473^***^	0.358^***^	0.355^***^	0.345^***^
CEDSE		0.224^***^		0.298^***^	0.213^***^	0.232^***^	0.196^***^
OS		0.051			0.094^*^	0.079	0.001
OCM		0.215^***^			0.258^***^	0.249^***^	0.252^***^
OS × CEDSE		0.186^**^				0.057	0.204^**^
OCM × CEDSE		0.023				0.002	0.005
OS × OCM		0.181^***^				−0.139^**^	−0.188^***^
OS × OCM × CEDSE		0.158^**^					0.192^**^
R^2^	0.333	0.482	0.385	0.448	0.503	0.516	0.532
ΔR^2^	—	0.149	0.329	0.063	0.055	0.013	0.016
F	153.107^***^	34.784^***^	8.982^***^	11.057^***^	12.490^***^	11.520^***^	11.795^***^

N = 308. Coefficients are standardized regression coefficients (β). CC = Career Crafting, PP = Proactive Personality, CEDSE = Career Exploration and Decision-Making Self-Efficacy, OS = Occupational Stigma, OCM = organizational career management. All interaction terms were mean-centered before analysis. Models 1 and 2 exclude control variables; Models 3 to 7 include all covariates. Categorical variables were dummy-coded, and ordinal variables were treated as continuous. Control coefficients are omitted for brevity. The baseline model with only control variables yielded R^2^ = 0.056, F = 0.898 (ns). ΔR^2^ represents the variance explained relative to the prior model. ^*^p < 0.05, ^**^p < 0.01, ^***^p < 0.001.

### Test of mediation

Mediation analysis was performed using the PROCESS macro (Model 4) with 10,000 bootstrap resamples and 95% bias-corrected confidence intervals. All demographic and occupational covariates, including gender (dummy coded), age group, education level, type of job (dummy coded), title level, job hierarchy, years of experience in occupation, years of work in organization, nature of hospital (dummy coded), and hospital level (dummy coded), were included as control variables. The results are presented in Table 4. The total effect of proactive personality on career crafting was significant (B = 0.650, SE = 0.053, 95% CI [0.547, 0.754]). After including the mediator (CEDSE), the direct effect of proactive personality on career crafting remained significant (B = 0.512, SE = 0.055, 95% CI [0.403, 0.621]), consistent with Hypothesis 1. The indirect association of proactive personality with career crafting via CEDSE was also significant (B = 0.138, Boot SE = 0.044, 95% Boot CI [0.066, 0.238]), accounting for 21.24% of the total effect (0.138/0.650). These results are consistent with Hypothesis 2.

**Table 4:** Results of total effect, direct effect, and indirect effect.

Variables/Path	β	B	SE	Bias-corrected confidence intervals (Boot 95% CI)	
				LLCI	ULCI
Total effect (Proactive personality → Career crafting)	0.601	0.650	0.053	0.547	0.754
Direct effect (Proactive personality → Career crafting, controlling for CEDSE)	0.473	0.512	0.055	0.403	0.621
Indirect effect (Proactive personality → CEDSE → Career crafting)	0.128	0.138	0.044	0.066	0.238

### Test of moderation

To examine the moderating role of occupational stigma between the CEDSE and career crafting relationship as proposed in H3a, we adopted PROCESS Model 1 with 10,000 bias-corrected bootstrap samples and 95% confidence intervals (CIs). All continuous predictors were mean-centered before analysis. The model controlled for gender, age group, education level, job type, professional title, job hierarchy, occupational tenure, organizational tenure, hospital nature and hospital level, with all categorical variables dummy-coded.

As shown in Table 5, CEDSE was positively associated with career crafting (B = 0.327, SE = 0.036, 95% CI [0.257, 0.398]), and occupational stigma also showed a positive association with career crafting (B = 0.221, SE = 0.047, 95% CI [0.128, 0.315]). The interaction term CEDSE × occupational stigma was marginally significant (B = 0.043, SE = 0.023, 95% CI [−0.003, 0.088], *p* = 0.065). Hypothesis H3a was not supported. For further descriptive analysis, simple slope tests were conducted at low (−1 SD), mean, and high (+1 SD) levels of occupational stigma. The positive linkage between CEDSE and career crafting remained significant across all occupational stigma levels: low occupational stigma (B = 0.276, SE = 0.048, 95% CI [0.182, 0.369]) and high occupational stigma (B = 0.379, SE = 0.043, 95% CI [0.294, 0.464]). The simple slopes were significant at both low and high levels, with a slightly larger coefficient at the high level. Given the marginal significance of the interaction, future studies with larger sample sizes or experimental designs are recommended to further verify this potential moderating pattern.

**Table 5:** Moderating effect of occupational stigma between CEDSE and career crafting.

Variables/Path	B	SE	95% CI	*p*
Dependent variable: career crafting (R^2^ = 0.347, *F* = 6.884)				
CEDSE	0.327	0.036	[0.257, 0.398]	<0.001
Occupational Stigma	0.221	0.047	[0.128, 0.315]	<0.001
Int1 (CEDSE × Occupational Stigma)	0.043	0.023	[−0.003, 0.088]	0.065
Low occupational stigma	0.276	0.048	[0.182, 0.369]	<0.001
High occupational stigma	0.379	0.043	[0.294, 0.464]	<0.001

To test whether the conditional association between CEDSE and career crafting at different levels of occupational stigma varies systematically with organizational career management, a three-way interaction analysis was conducted using Model 18 of the PROCESS macro (10,000 bootstrap samples, 95% bias-corrected confidence intervals). All continuous predictors were mean-centered prior to analysis. The model controlled for a comprehensive set of demographic and occupational covariates, all categorical variables were dummy coded with reference groups as specified in the measures section. As shown in Table 6, the three-way interaction “CEDSE×occupational stigma × organizational career management” was significant (B = 0.050, SE = 0.016, 95% CI [0.018, 0.081], *p* = 0.002 < 0.01), consistent with the hypothesis that the moderating pattern of occupational stigma on the CEDSE and career crafting relationship varies with organizational career management. Thus, Hypothesis H3b was supported.

**Table 6:** Moderating role of organizational career management.

Variables/Path	B	SE	95% CI	*p*
Dependent variable: career crafting (R^2^ = 0.532, *F* = 11.795)				
Proactive personality (PP)	0.374	0.058	[0.259, 0.489]	<0.001
CEDSE	0.136	0.040	[0.058, 0.213]	<0.001
Occupational stigma (OS)	0.001	0.049	[−0.096, 0.098]	0.980
Organizational career management (OCM)	0.359	0.075	[0.212, 0.507]	<0.001
Int1 (CEDSE × OS)	0.093	0.032	[0.030, 0.156]	0.004
Int2 (CEDSE × OCM)	0.003	0.028	[−0.052, 0.058]	0.917
Int3 (OS × OCM)	−0.169	0.047	[−0.261, −0.076]	<0.001
Int4 (CEDSE × OS × OCM)	0.050	0.016	[0.018, 0.081]	0.002
Low OS, Low OCM	0.069	0.048	[−0.025, 0.164]	0.150
Low OS, High OCM	−0.022	0.079	[−0.177, 0.133]	0.779
Mean OS, Low OCM	0.133	0.038	[0.058, 0.209]	<0.001
Mean OS, High OCM	0.138	0.051	[0.037, 0.239]	0.008
High OS, Low OCM	0.197	0.050	[0.099, 0.296]	<0.001
High OS, High OCM	0.298	0.065	[0.170, 0.425]	<0.001

**Figure 2:**
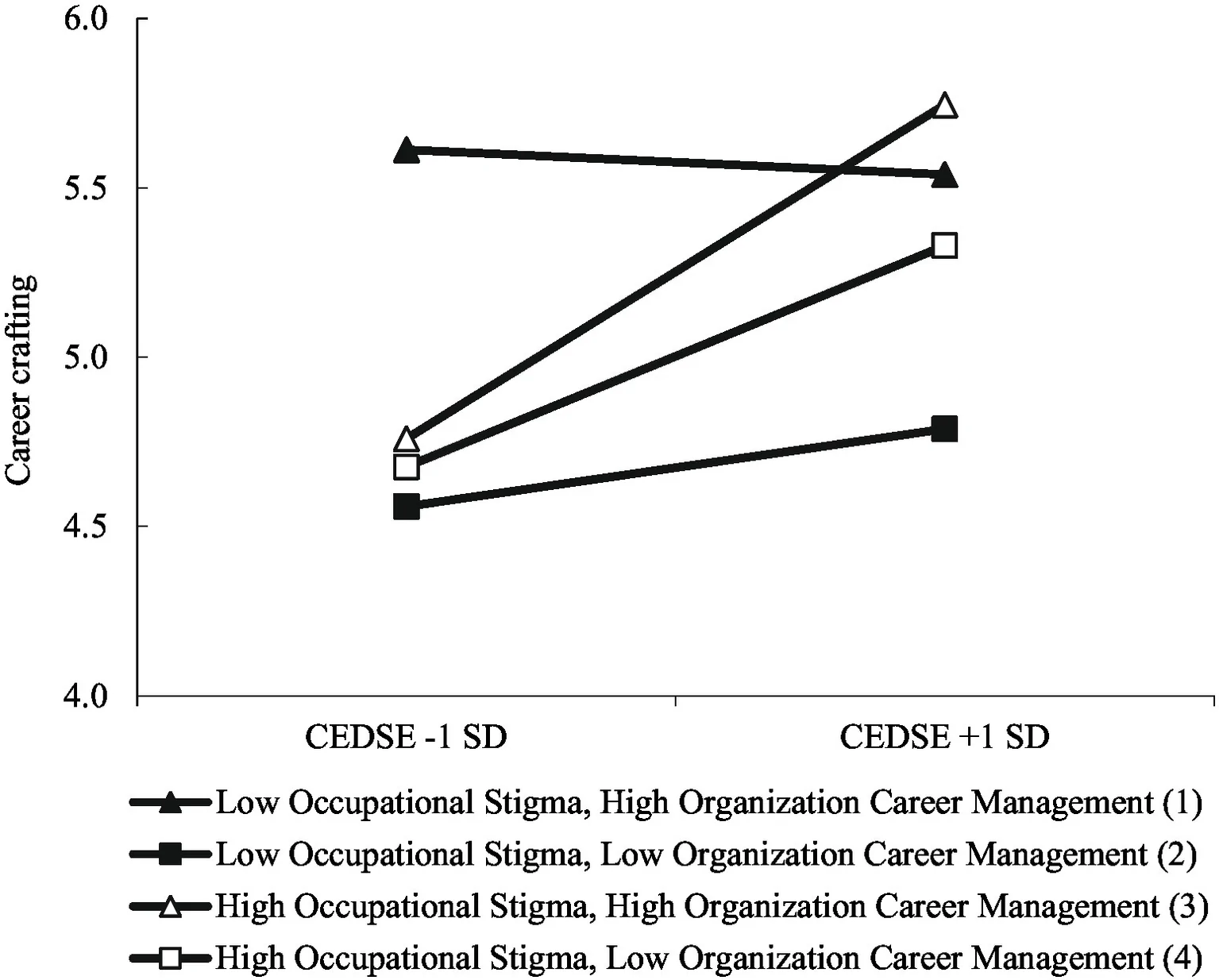
The three-way interaction effect of CEDSE × occupational stigma × organizational career management on career crafting.

**Figure 3:**
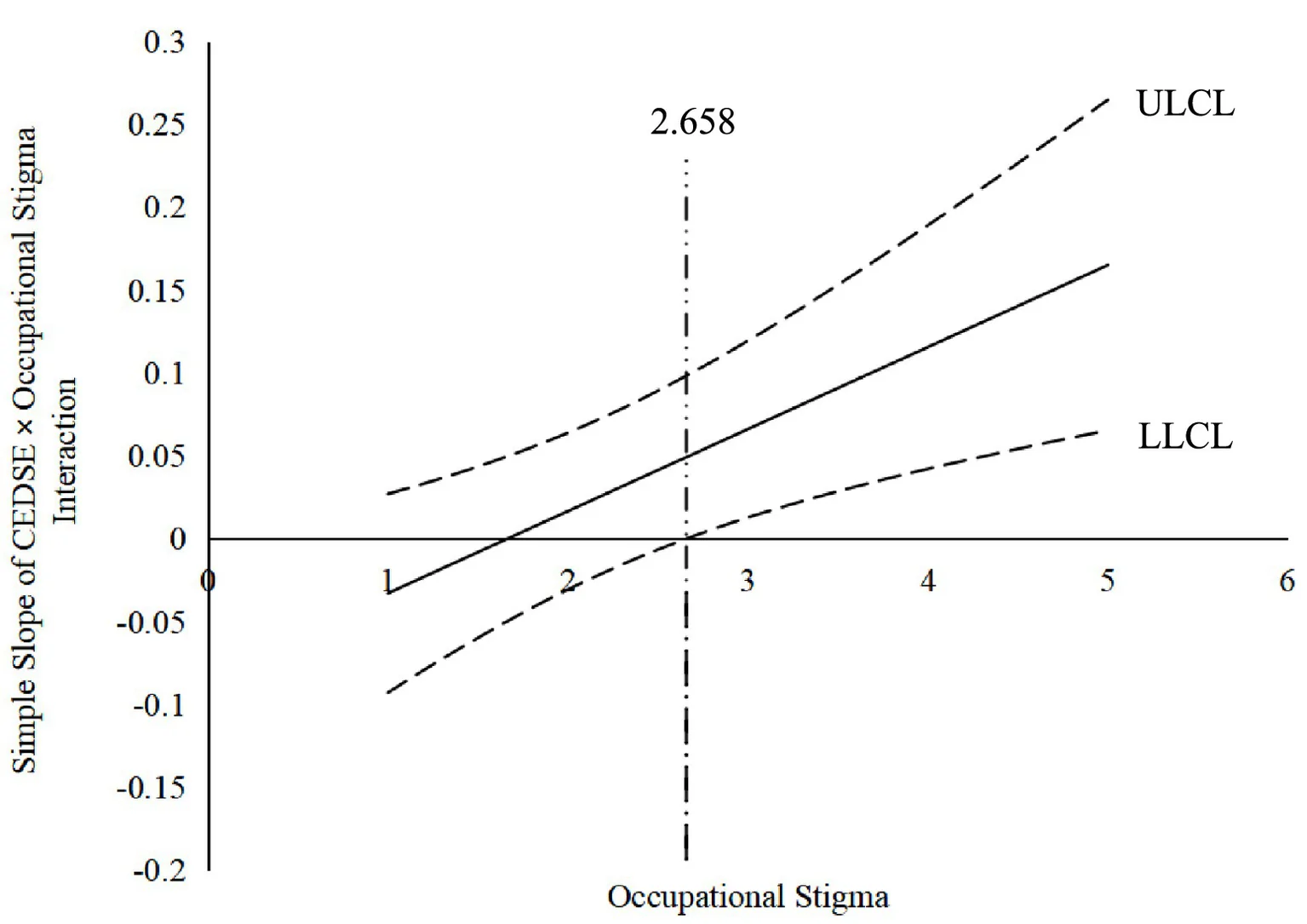
The moderating effect of organizational career management on the moderating role of occupational stigma. The variable values in the figure have been converted back to the original scale from mean-centered data for ease of interpretation.

Figure 2 displays the three-way interaction, illustrating that the moderating pattern of occupational stigma on the CEDSE and career crafting relationship is more pronounced at higher levels of organizational career management. To probe the nature of this three-way interaction, we examined the conditional two-way interaction between CEDSE and occupational stigma at low (−1 SD) and high (+1 SD) levels of organizational career management. The CEDSE×occupational stigma interaction was significant at both low organizational career management (B = 0.053, *p* = 0.037 < 0.05) and high organizational career management (B = 0.132, *p* = 0.002 < 0.01), with the pattern more pronounced at higher organizational career management. Table 6 further reports the conditional associations of CEDSE with career crafting at combinations of occupational stigma and organizational career management. The positive association between CEDSE and career crafting was most pronounced when both occupational stigma and organizational career management were high (B = 0.298, SE = 0.065, 95% CI [0.170, 0.425]) and non-significant when occupational stigma was low regardless of organizational career management level. Johnson-Neyman analysis indicated that the CEDSE×occupational stigma interaction reached statistical significance when organizational career management exceeded −0.880 (standardized score, corresponding to a raw score of 2.658, covering 88.96% of the sample). Figure 3 illustrates how organizational career management strengthens the moderating role of occupational stigma. As organizational career management increased, the interactive relationship between CEDSE and occupational stigma on career crafting became progressively stronger, a pattern consistent with organizational career management serving as an enabling resource that is associated with a more pronounced moderating pattern of occupational stigma. Consistent with our theoretical framework, organizational career management functions as a resource-providing enabler instead of a stress buffer: high organizational resources help individuals transform stigma pressure into proactive career actions.

The moderated moderated mediation analysis (PROCESS Model 18, 10,000 bootstrap samples) examined whether the indirect effect of proactive personality on career crafting via CEDSE varies systematically with the combined levels of occupational stigma and organizational career management. As shown in Table 7, the index of moderated moderated mediation was 0.033, with a 95% bootstrap confidence interval of [0.004, 0.085], excluding zero. This finding is consistent with Hypothesis H3c, suggesting that the indirect effect of proactive personality on career crafting through CEDSE may be contingent upon the interactive influence of occupational stigma and organizational career management.

**Table 7:** Moderated mediation effects.

Occupational stigma	Organizational career management	B	SE	95% CI
Low	Low	0.046	0.039	[−0.009, 0.144]
Low	High	−0.015	0.064	[−0.153, 0.104]
Mean	Low	0.089	0.034	[0.032, 0.164]
Mean	High	0.092	0.049	[−0.004, 0.191]
High	Low	0.132	0.052	[0.029, 0.235]
High	High	0.199	0.085	[0.048, 0.376]
Index of moderated moderated mediation		0.033	0.021	[0.004, 0.085]

The conditional indirect effects revealed a clear pattern. Under low occupational stigma, the indirect effect was non-significant across all levels of organizational career management (all confidence intervals included zero). Under mean occupational stigma, the indirect effect was significant at low organizational career management (effect = 0.089, 95% CI [0.032, 0.164]) and approached significance at high organizational career management (effect = 0.092, 95% CI [−0.004, 0.191]). Under high occupational stigma, the indirect effect was significant and showed an increasing trend as organizational career management increased: from 0.132 at low organizational career management (95% CI [0.029, 0.235]) to 0.199 at high organizational career management (95% CI [0.048, 0.376]). The largest indirect effect was observed when both occupational stigma and organizational career management were high (effect = 0.199), a pattern consistent with the theoretical expectation that the mediating pathway may be most salient when high contextual threat coincides with high organizational support.

### Robustness checks

After completing the core hypothesis tests, we conducted a comprehensive set of robustness checks to evaluate the reliability of our findings.

1. Model specification. As shown in Table 6, the effect of proactive personality on career crafting was significant both with and without control variables (without controls: *β* = 0.577, *p* < 0.001; with controls: β = 0.601, *p* < 0.001). The three-way interaction was also consistent across these specifications (without controls: β = 0.158, *p* < 0.01; with controls: *β* = 0.192, *p* < 0.01), confirming that our findings are not artifacts of covariate inclusion.
2. Multicollinearity. All VIF values in the full model were well below the conventional threshold of 10, ranging from 1.16 to 8.95 for control variables and from 1.34 (organizational career management × CEDSE) to 2.98 (occupational stigma × CEDSE) for interaction terms. All tolerance values exceeded 0.1 (range: 0.11–0.86). Specifically, for the study variables, VIF values were 1.74 (proactive personality), 1.95 (CEDSE), 1.62 (occupational stigma), 1.66 (organizational career management), 2.98 (occupational stigma × CEDSE), 1.34 (organizational career management × CEDSE), 1.64 (occupational stigma × organizational career management), and 2.27 (occupational stigma × organizational career management × CEDSE). These results indicate no serious multicollinearity.
3. Influential cases. Seven cases with studentized residuals exceeding ±3 were identified (maximum Cook’s distance = 0.312, all < 1). Excluding these cases did not alter the significance of the three-way interaction (β = 0.114, *p* = 0.047), confirming that no individual observations unduly influenced the results.
4. Heteroscedasticity-consistent standard errors. Given our moderate sample size (*N* = 308), we adopted the HC2 estimator, which provides optimal Type I error control for samples in this range (Long and Ervin, 2000). The three-way interaction remained significant under HC2 (B = 0.050, HC2 SE = 0.024, *p* = 0.043), consistent with the OLS estimates.
5. Alternative model comparisons. Three alternative models were tested to further evaluate our hypothesized framework. A simpler moderation model with occupational stigma as the predictor and organizational career management as the moderator (occupational stigma × organizational career management → career crafting) yielded a non-significant interaction (B = −0.043, SE = 0.045, *p* = 0.341) and explained only 35.8% of the variance in career crafting, substantially less than our hypothesized model (R^2^ = 0.532, ΔR^2^ = 0.174). A competitive model specifying CEDSE as the antecedent and proactive personality as the moderator produced a significant CEDSE × proactive personality interaction (B = 0.058, SE = 0.024, *p* = 0.017), but with lower overall fit (R^2^ = 0.459 vs. 0.532). A reverse-path model (career crafting → proactive personality → CEDSE) yielded a significant indirect effect (B = 0.185, BootSE = 0.073, 95% CI [0.063, 0.347]), consistent with known cross-sectional reciprocity between self-efficacy and proactive behavior, and explained 36.3% of the variance in CEDSE. Taken together, these comparisons indicate that our hypothesized conditional process model provides superior explanatory power and theoretical coherence relative to plausible alternatives.

In summary, all key findings remained consistent across the full range of robustness checks, demonstrating the reliability of our empirical results.

## Discussion

### Theoretical implications

This study contributes to theoretical understanding by integrating the personal agency perspective from Social Cognitive Career Theory with the contextual stress perspective from cognitive appraisal theory of stress. The findings offer three primary theoretical implications, pointing toward an integrated framework.

First, this study addresses the central question regarding the origins of proactive career behaviors by examining the theoretical pathway linking dispositional traits to individual actions. The findings provide preliminary cross-sectional evidence that proactive personality may serve as a distal factor associated with career crafting, complementing the specific motivational or conditional factors emphasized in prior literature [e.g., self-commitment (Alarifi et al., 2024), career-adaptive resources (Nalis et al., 2022), family motivation (Ge et al., 2025), and career calling (Ni et al., 2024)]. This adds to research on the antecedents of career crafting and responds to scholarly calls to examine core dispositional traits (Ge et al., 2023). More importantly, the research presents tentative patterns suggesting that CEDSE may act as a key linkage through which a broad dispositional tendency correlates with purposeful, self-directed career crafting. Specifically, drawing on social cognitive career theory and its career self-management model (Lent and Brown, 2013; Lent et al., 1994), proactive personality is positively associated with individuals’ perceived self-competence and coping efficacy in managing career tasks and navigating career transitions (Kim and Park, 2017; Park, 2015; Acar, 2022). In turn, higher CEDSE correlates with greater optimism and perseverance, which may be associated with engagement in proactive career behaviors (Bandura, 2001), even when faced with potential obstacles or resistance (Axtell et al., 2000). Thus, individuals with a proactive disposition, together with a stronger belief in their career management capabilities, tend to engage in deliberate career crafting more frequently. These observed patterns align with the core proposition of the career self-management model of social cognitive career theory within the context of career crafting (Lent and Brown, 2013; Lent et al., 1994). Accordingly, this exploratory analysis enriches the understanding of personal agency associated with proactive career development.

Second, the two-way interaction between CEDSE and occupational stigma was only marginally significant, whereas the three-way interaction of CEDSE, occupational stigma, and organizational career management was statistically significant. This pattern indicates that the moderating role of occupational stigma does not operate independently; instead, it is contingent upon the level of organizational career management. This finding does not contradict the well-documented adverse impacts of occupational stigma on medical professionals’ well-being, burnout, and turnover intentions (Wang et al., 2020; Sun et al., 2021). Occupational stigma remains fundamentally a harmful social phenomenon. Instead, it points to an additional conditional coping pattern that coexists with these negative outcomes. These cross-sectional observations yield new insights into cognitive appraisal theory of stress by illustrating how a contextual stressor and a contextual resource jointly correlate with the translation of self-efficacy into proactive behavior. Specifically, when medical professionals appraise occupational stigma as a threat to their professional identity (Goffman, 1963; Dovidio et al., 2000), this appraisal may be linked to proactive identity work, with career crafting serving as one strategic behavior to reassert autonomy and reshape the career path in defense of the professional self (Petriglieri, 2011; Caza et al., 2018). Consistent with the descriptive pattern from simple slope analysis, a stronger perceived threat tended to show a numerically larger coefficient for the CEDSE and career crafting relationship; however, the two-way interaction did not reach statistical significance, so this trend should be interpreted with caution and warrants replication.

Moreover, the results further clarify the role of organizational career management within the secondary appraisal process. Crucially, organizational career management does not merely buffer the negative effects of stigma; rather, it may provide tangible resources such as career guidance, skill development, and clearer pathways (Yildiz et al., 2024; Bagdadli and Gianecchini, 2019), which are associated with enhanced perceived capacity to cope with identity-related threats. When organizational career management is high, the moderating trend of occupational stigma on the CEDSE and career crafting linkage becomes stronger. Conversely, under low organizational career management, the same stigmatizing pressure correlates with a weaker moderating pattern, potentially due to perceived higher risk and insufficient support to translate CEDSE into action (Canboy et al., 2023). Thus, this study extends the application of cognitive appraisal theory of stress by describing how a contextual resource correlates with behavioral outcomes arising from threat appraisal, offering a more integrated perspective for understanding proactive career crafting in stigmatized work contexts. Nonetheless, this conditional coping pattern does not negate the overall damaging effects of occupational stigma, which remain a critical concern for healthcare organizations.

Third, the observed patterns are consistent with a moderated moderated mediation structure, showing that the indirect linkage of proactive personality to career crafting via CEDSE varies across the combined levels of occupational stigma and organizational career management. This conditional pattern suggests that under specific circumstances, when individuals hold high self-efficacy and receive strong organizational support, stigma-related pressure may be associated with constructive coping behaviors. This exploratory finding advances theoretical integration by delineating potential boundary conditions for the core social cognitive career theory pathway through the lens of cognitive appraisal theory of stress, suggesting that personal agency may have the strongest association with proactive career behavior when high contextual threat coincides with high organizational support. While individual agency plays a vital role in sustaining careers amid societal changes and uncertain career paths (Parker and Liao, 2016; De Vos et al., 2020), our correlational results also point to the indispensable role of organizations (De Vos et al., 2019; Baruch and Sullivan, 2022). By describing how organizational career management and occupational stigma may jointly condition the indirect linkage of proactive personality to career crafting through CEDSE, this study aligns with prior research reporting a positive correlation between career crafting and organizational career management (Lee et al., 2021), as well as studies linking developmental human resource practices to proactive career behaviors (Yang et al., 2024). Significantly, the findings suggest that organizational career management and individual career self-management can function as complementary rather than opposing forces. Consequently, this cross-sectional research provides preliminary evidence for a contextually grounded, integrative framework that synthesizes career self-management and organizational career management perspectives, characterizing the interactive associations between personal agency and organizational support in proactive career development.

### Practical implications

Although this cross-sectional correlational study cannot establish causal relationships, it points to several promising directions for future research related to career crafting among medical professionals, while highlighting the interplay between individual and organizational factors.

First, the results are consistent with the view that proactive personality is positively associated with career crafting through CEDSE. While proactive personality, as a relatively stable personality trait, is difficult to modify (Bateman and Crant, 1993), the identification of CEDSE as a potential mediating mechanism suggests that CEDSE-targeted interventions are a valuable direction for follow-up research. As a form of psychological capital, CEDSE is not as malleable as transient moods but is more amenable to development than stable personality traits (Abid et al., 2021). Numerous prior studies have confirmed that career interventions can correlate with changes in career-related self-efficacy (Falco and Summers, 2017; Glessner et al., 2017). Therefore, future research could examine whether interventions aimed at fostering CEDSE are associated with increased career crafting, especially among medical professionals with lower levels of proactive personality.

Drawing on Social Cognitive Career Theory, which defines four core sources of self-efficacy: mastery experiences, vicarious learning, verbal persuasion, and physiological and emotional arousal (Lent et al., 1994), as well as validated career intervention components from prior literature (Brown et al., 2003; Chiesa et al., 2016), future CEDSE-related research may focus on the following research dimensions: (1) structured written tasks designed to build mastery experiences based on successful career cases; (2) vicarious learning via role modeling and mentorship; (3) personalized feedback and encouragement through career coaching; (4) skill training for career-related emotion regulation. The practical effectiveness of these approaches remains to be verified through formal intervention studies.

Second, the findings suggest organizational career management is positively associated with career crafting, and this linkage tends to be stronger for individuals exposed to occupational stigma. Prior research indicates that medical professionals often lack dedicated career management support from organizations and supervisors (van Leeuwen, 2023). Combined with our observations, organizational career management may carry unique value for employees working under stigmatized conditions. Given that occupational stigma exerts widespread adverse impacts on individuals and the broader healthcare system (Wang et al., 2020; Lien et al., 2016), exploring organizational career management’s role in such contexts is a meaningful research agenda.

Existing literature has proposed a set of strategies to help individuals rebuild professional identity amid occupational stigma (Flensborg Jensen, 2017; Grandy and Mavin, 2011; Brewis and Godfrey, 2017; Slutskaya et al., 2016; Ashforth et al., 2017). Future research could explore whether organizational practices aligned with these identity-building approaches correlate with proactive career behaviors. For example, subsequent studies may investigate the relationships between professional identity guidance, vocational skill training, mentorship networks and non-clinical career programs and career crafting among medical staff (Wu and Liu, 2022; Westein et al., 2019).

Finally, the findings indicate that exploring the combination of CEDSE development research and organizational career management research is a promising direction. Instead of treating individual psychological development and organizational career support as separate research topics, future work could examine the synergistic effects of integrating self-efficacy coaching, stigma-coping workshops, career navigation services and mentorship within a unified career development framework.

### Limitations and future research

Several limitations of this study need to be acknowledged, along with corresponding directions for future research.

First, this study adopted a cross-sectional design. Career crafting is a long-term behavioral tendency that involves ongoing planning for future career development (van Leeuwen et al., 2021). A single snapshot of data cannot fully capture individuals’ long-term career management efforts. Furthermore, consistent with career construction theory, adaptive personal resources and behavioral outcomes often form dynamic interconnections (Savickas, 2013). Prior empirical evidence has documented the bidirectional association between CEDSE and career exploratory behaviors (Rogers and Creed, 2011; El-Hassan and Ghalayini, 2020). When individuals engage in career crafting and gain positive experiences, these experiences may also be associated with elevated career self-efficacy. Therefore, future studies could adopt multi-wave longitudinal designs to clarify the temporal sequence of these interrelated variables.

Second, the generalizability of the findings may be limited by sample characteristics. The sample is predominantly female, which aligns with the gender distribution of frontline medical staff in China but warrants caution in direct extrapolation to other groups. Perceptions of occupational stigma and career-related behaviors may differ across genders due to varied life experiences and social expectations (Balakhtar et al., 2024; Amin et al., 2025). In addition, all participants were recruited from 12 grassroots healthcare institutions, which further constrains the scope of generalization. Future research could replicate this moderated moderated mediation model in gender-balanced samples, male-dominated medical departments such as surgical disciplines, leadership groups, and healthcare systems in other cultural contexts, to explore potential boundary conditions and improve external validity.

Third, all data in this study were collected via self-report questionnaires, which may introduce common method variance and affect result interpretation (Fuller and Marler, 2009; Podsakoff et al., 2012). As prior research indicated, same-source data may inflate the observed associations between proactive personality and behavioral outcomes. To address this issue, future research could adopt multi-source data collection: for instance, combining supervisor and coworker evaluations to measure career crafting. The assessment of career crafting can also incorporate interview ratings and scenario-based measurements (Frese et al., 1997; Parker et al., 2006; Bledow and Frese, 2009). For the measurement of occupational stigma, researchers may supplement questionnaire data with archival materials and public media reports (Marsh et al., 2004).

Fourth, two additional methodological limitations deserve attention. The occupational stigma scale used in this study was adapted from a scale originally developed for call center employees. Although we invited experts to conduct qualitative content evaluation for medical populations, quantitative content validity indices such as I-CVI and S-CVI were not calculated, leaving room for further scale validation in medical settings. Additionally, the two-way interaction between CEDSE and occupational stigma only reached marginal significance in this study. This observed pattern requires re-verification with larger samples in follow-up research.

Finally, this study only examined proactive personality as the core dispositional antecedent. As noted by Parker et al. (2010), different personality traits often interact and form complementary or synergistic relationships. For example, high proactivity without sufficient cognitive deliberation may be associated with less adaptive career behaviors. The combined associations of multiple personality traits with career crafting remain underexplored. Moreover, this study mainly focuses on individual self-reported career behaviors, while the long-term career outcomes associated with career crafting are not examined. Drawing on existing studies on proactivity and career self-management (Lent and Brown, 2013; Parker et al., 2010), future research could explore the joint contributions of multiple personality traits, as well as the relationship between career crafting and objective career outcomes.

## Conclusion

This study examined an integrated career crafting model by combining career self-management model and organizational career management perspectives among medical professionals. Grounded in Social Cognitive Career Theory, the model positions career exploration and decision-making self-efficacy as a key mechanism linking proactive personality to career crafting. Drawing on cognitive appraisal theory of stress, the framework further suggests a two-stage moderating structure: occupational stigma moderates the relationship between the career exploration and decision-making self-efficacy and career crafting, and this moderating pattern is further conditioned by organizational career management support. Collectively, these pathways form a comprehensive moderated moderated mediation framework. The results suggest that the indirect association between proactive personality and career crafting via career exploration and decision-making self-efficacy varies depending on the combined influence of occupational stigma and organizational career management support. This exploratory analysis provides preliminary evidence for how individual dispositional traits may relate to proactive career behaviors through self-regulatory processes, which are jointly shaped by contextual stressors and organizational resources.

## Data Availability

The original contributions presented in the study are included in the article/Supplementary material, further inquiries can be directed to the corresponding author.

## References

[ref1] Abid G. Arya B. Arshad A. Ahmed S. Farooqi S. (2021). Positive personality traits and self-leadership in sustainable organizations: mediating influence of thriving and moderating role of proactive personality. Sustainable Prod. Consumption 25, 299–311. doi: 10.1016/j.spc.2020.09.005

[ref2] Acar S. (2022). The association of career talent self-efficacy, positive future expectations and personal growth initiative. Psychoeduc. Res. Rev. 11, 246–253. doi: 10.52963/PERR_Biruni_V11.N1.15

[ref3] Aiken L. S. West S. G. (1991). Multiple Regression: Testing and Interpreting Interactions. Newbury Park, CA: Sage.

[ref4] Akkermans J. Hirschi A. (2023). Career proactivity: conceptual and theoretical reflections. Appl. Psychol. 72, 199–204. doi: 10.1111/apps.12444

[ref5] Alarifi A. Bajaba S. Basahal A. (2024). From self-commitment to career crafting: the mediating role of career adaptability and the moderating role of job autonomy. Acta Psychol. 248:104387. doi: 10.1016/j.actpsy.2024.104387, 38968809

[ref6] Amin S. M. Demerdash D. E. Othman A. A. Zoromba M. A. El-Gazar H. E. Atta M. H. R. . (2025). The mediating role of professional identity in the relationship between gender misconceptions and occupational stigma among male nursing students. BMC Nurs. 24:930. doi: 10.1186/s12912-025-03552-5, 40676586 PMC12269126

[ref7] Anderson J. C. Gerbing D. W. (1988). Structural equation modeling in practice: a review and recommended two-step approach. Psychol. Bull. 103, 411–423. doi: 10.1037/0033-2909.103.3.411

[ref8] Arnold J. (1997). Managing Careers Into the 21st Century. London: Paul Chapman Publishing.

[ref9] Ashforth B. E. (2018). Stigma and legitimacy: two ends of a single continuum or different continua altogether? J. Manage. Inquiry 28, 22–30. doi: 10.1177/1056492618790900

[ref10] Ashforth B. E. Kreiner G. E. (1999). "how can you do it?": dirty work and the challenge of constructing a positive identity. Acad. Manag. Rev. 24, 413–434. doi: 10.2307/259134, 32922005 PMC7455605

[ref11] Ashforth B. E. Kreiner G. E. Clark M. A. Fugate M. (2017). Congruence work in stigmatized occupations: a managerial lens on employee fit with dirty work. J. Organ. Behav. 38, 1260–1279. doi: 10.1002/job.2201

[ref12] Axtell C. M. Holman D. J. Unsworth K. L. Wall T. D. Waterson P. E. Harrington E. (2000). Shopfloor innovation: facilitating the suggestion and implementation of ideas. J. Occup. Organ. Psychol. 73, 265–285. doi: 10.1348/096317900167029

[ref13] Bagdadli S. Gianecchini M. (2019). Organizational career management practices and objective career success: a systematic review and framework. Hum Resour Manage Rev. 29, 353–370. doi: 10.1016/j.hrmr.2018.08.001, 38826717

[ref14] Bakker A. B. Tims M. Derks D. (2012). Proactive personality and job performance: the role of job crafting and work engagement. Hum. Relat. 65, 1359–1378. doi: 10.1177/0018726712453471

[ref15] Balakhtar V. Bondarchuk O. Kazakova S. (2024). Career crafting literature meta-review. Multidiscipl Sci J. 6:2024ss0741. doi: 10.31893/multiscience.2024ss0741

[ref16] Bandura A. (1978). The self system in reciprocal determinism. Am. Psychol. 33, 344–358. doi: 10.1037/0003-066X.33.4.344

[ref17] Bandura A. (1997). Self-Efficacy: The Exercise of Control. New York: W H Freeman/Times Books/Henry Holt & Co.

[ref18] Bandura A. (2001). Social cognitive theory: an agentic perspective. Annu. Rev. Psychol. 52, 1–26. doi: 10.1146/annurev.psych.52.1.1, 11148297

[ref19] Bandura A. (2006). “Guide for constructing self-efficacy scales,” in Self-Efficacy Beliefs of Adolescents, eds. Urdan T. Pajares F. (Greenwich: Information Age Publishing), 307–337.

[ref20] Baruch Y. Sullivan S. E. (2022). The why, what and how of career research: a review and recommendations for future study. Career Dev. Int. 27, 135–159. doi: 10.1108/CDI-10-2021-0251

[ref21] Bateman T. S. Crant J. M. (1993). The proactive component of organizational behavior: a measure and correlates. J. Organ. Behav. 14, 103–118. doi: 10.1002/job.4030140202

[ref22] Betz N. E. Klein K. L. Taylor K. M. (1996). Evaluation of a short form of the career decision-making self-efficacy scale. J. Career Assess. 4, 47–57. doi: 10.1177/106907279600400103

[ref23] Bindl U. K. Parker S. K. (2011). “Proactive work behavior: forward-thinking and change-oriented action in organizations,” in APA Handbook of Industrial and Organizational Psychology, Vol 2: Selecting and Developing Members for the Organization, ed. Zedeck S. (Washington, DC: American Psychological Association), 567–598.

[ref24] Bledow R. Frese M. (2009). A situational judgment test of personal initiative and its relationship to performance. Pers. Psychol. 62, 229–258. doi: 10.1111/j.1744-6570.2009.01137.x

[ref25] Brass D. J. (2001). “Social capital and organizational leadership,” in The Nature of Organizational Leadership: Understanding the Performance Imperatives Confronting Today's Leaders, eds. Zaccaro S. J. Klimoski R. J. (San Francisco: Jossey-Bass), 132–152.

[ref26] Brewis J. Godfrey R. (2017). 'Never call me a mercenary': identity work, stigma management and the private security contractor. Organization 25, 335–353. doi: 10.1177/1350508417710830

[ref27] Brown S. D. Ryan Krane N. E. Brecheisen J. Castelino P. Budisin I. Miller M. . (2003). Critical ingredients of career choice interventions: more analyses and new hypotheses. J. Vocat. Behav. 62, 411–428. doi: 10.1016/S0001-8791(02)00052-0

[ref28] Canboy B. Tillou C. Barzantny C. Güçlü B. Benichoux F. (2023). The impact of perceived organizational support on work meaningfulness, engagement, and perceived stress in France. Eur. Manag. J. 41, 90–100. doi: 10.1016/j.emj.2021.12.004

[ref29] Caza B. B. Vough H. Puranik H. (2018). Identity work in organizations and occupations: definitions, theories, and pathways forward. J. Organ. Behav. 39, 889–910. doi: 10.1002/job.2318

[ref30] Chiesa R. Massei F. Guglielmi D. (2016). Career decision-making self-efficacy change in Italian high school students. J. Couns. Dev. 94, 210–224. doi: 10.1002/jcad.12077

[ref31] Chifor R. I. Oprea B. (2023). Romanian version of the career crafting assessment: psychometric properties. J. Career Assess. 31, 717–736. doi: 10.1177/10690727231163322

[ref32] Cinamon R. G. (2006). Anticipated work-family conflict: effects of gender, self-efficacy, and family background. Career Dev. Q. 54, 202–215. doi: 10.1002/j.2161-0045.2006.tb00152.x

[ref33] Cohen J. Cohen P. West S. G. Aiken L. S. (2003). Applied multiple Regression/Correlation Analysis for the Behavioral Sciences. 3rd Edn Mahwah, NJ: Lawrence Erlbaum.

[ref34] Coleman J. S. (1988). Social capital in the creation of human capital. Am. J. Sociol. 94, S95–S120. doi: 10.1086/228943

[ref35] Crant J. M. (2000). Proactive behavior in organizations. J. Manage. 26, 435–462. doi: 10.1177/014920630002600304

[ref36] De Vos A. Akkermans J. Van der Heijden B. (2019). “From occupational choice to career crafting,” in The Routledge Companion to Career Studies, eds. Gunz H. Lazarova M. Mayrhofer W. (London: Routledge), 128–142.

[ref37] De Vos A. Van der Heijden B. I. J. M. Akkermans J. (2020). Sustainable careers: towards a conceptual model. J. Vocat. Behav. 117:103196. doi: 10.1016/j.jvb.2018.06.011, 38826717

[ref38] Dovidio J. F. Major B. Crocker J. (2000). “Stigma: introduction and overview,” in The Social Psychology of Stigma, eds. Heatherton T. F. Kleck R. E. Hebl M. R. Hull J. G. (New York: Guilford Press), 1–28.

[ref39] El-Hassan K. Ghalayini N. (2020). Parental attachment bonds, dysfunctional career thoughts and career exploration as predictors of career decision-making self-efficacy of grade 11 students. Br. J. Guid. Couns. 48, 597–610. doi: 10.1080/03069885.2019.1645296

[ref40] Falco L. D. Summers J. J. (2017). Improving career decision self-efficacy and STEM self-efficacy in high school girls: evaluation of an intervention. J. Career Dev. 46, 62–76. doi: 10.1177/0894845317721651

[ref41] Fang G. Cao H. (2020). State versus private provision: how does China's market-oriented reform affect healthcare delivery? Econ. Transit. Inst. Change 28, 381–411. doi: 10.1111/ecot.12239

[ref42] Flensborg Jensen M. C. (2017). Gender stereotypes and the reshaping of stigma in rehabilitative eldercare. Gend. Work. Organ. 24, 656–674. doi: 10.1111/gwao.12191

[ref44] Frese M. Fay D. (2001). Personal initiative: an active performance concept for work in the 21st century. Res. Organ. Behav. 23, 133–187. doi: 10.1016/S0191-3085(01)23005-6

[ref45] Frese M. Fay D. Hilburger T. Leng K. Tag A. (1997). The concept of personal initiative: operationalization, reliability and validity in two German samples. J. Occup. Organ. Psychol. 70, 139–161. doi: 10.1111/j.2044-8325.1997.tb00639.x

[ref46] Frese M. Garst H. Fay D. (2007). Making things happen: reciprocal relationships between work characteristics and personal initiative in a four-wave longitudinal structural equation model. J. Appl. Psychol. 92, 1084–1102. doi: 10.1037/0021-9010.92.4.1084, 17638467

[ref47] Fuller B. Marler L. E. (2009). Change driven by nature: a meta-analytic review of the proactive personality literature. J. Vocat. Behav. 75, 329–345. doi: 10.1016/j.jvb.2009.05.008

[ref48] Ge X. Gao L. Xiong R. Zhang Q. Yu H. (2025). Homefront fuel for career growth: the role of career crafting and work-life balance. Career Dev. Int. 30, 345–362. doi: 10.1108/CDI-01-2024-0007

[ref49] Ge X. Gao L. Yu H. (2023). A new construct in career research: career crafting. Behav Sci (Basel). 13:1. doi: 10.3390/bs13010049, 36661621 PMC9854777

[ref50] General Office of the Central Committee of the Communist Party of China Opinions on further improving the healthcare service system (2023) Available onilne at: http://www.gov.cn/zhengce/2023-03/23/content_5748063.htm [Accessed May 31, 2026].

[ref51] Glessner K. Rockinson-Szapkiw A. J. Lopez M. L. (2017). "yes, I can": testing an intervention to increase middle school students' college and career self-efficacy. Career Dev. Q. 65, 315–325. doi: 10.1002/cdq.12110

[ref52] Goffman E. (1963). “Embarrassment and social organization,” in Identity and Anxiety: Survival of the Person in Mass Society, ed. Stein M. B. (Glencoe: Free Press), 541–548.

[ref53] Grandy G. Mavin S. (2011). Occupational image, organizational image and identity in dirty work: intersections of organizational efforts and media accounts. Organization 19, 765–786. doi: 10.1177/1350508411422582

[ref54] Griffin M. A. Parker S. K. Mason C. M. (2010). Leader vision and the development of adaptive and proactive performance: a longitudinal study. J. Appl. Psychol. 95, 174–182. doi: 10.1037/a0017263, 20085414

[ref55] Hair J. F. Risher J. J. Sarstedt M. Ringle C. M. (2019). When to use and how to report the results of PLS-SEM. Eur. Bus. Rev. 31, 2–24. doi: 10.1108/EBR-11-2018-0203

[ref56] Hakimi I. (2020). The role of proactive personality on job engagement by mediating work meaning. Posit Psychol Res. 6, 35–48. doi: 10.22108/PPLS.2020.122353.1915

[ref57] Hayes A. F. (2013). Introduction to Mediation, Moderation, and Conditional Process Analysis: A Regression-Based Approach. New York: Guilford Press.

[ref58] Hughes E. C. (1970). The humble and the proud: the comparative study of occupations. Sociol. Q. 11, 147–156. doi: 10.1111/j.1533-8525.1970.tb01440.x

[ref59] Kaiser C. R. Major B. McCoy S. K. (2004). Expectations about the future and the emotional consequences of perceiving prejudice. Personal. Soc. Psychol. Bull. 30, 173–184. doi: 10.1177/0146167203259927, 15030632

[ref60] Kanfer R. Wanberg C. R. Kantrowitz T. M. (2001). Job search and employment: a personality-motivational analysis and meta-analytic review. J. Appl. Psychol. 86, 837–855. doi: 10.1037/0021-9010.86.5.837, 11596801

[ref61] Kim H. S. Park I. J. (2017). Influence of proactive personality on career self-efficacy. J. Employ. Couns. 54, 168–182. doi: 10.1002/joec.12065

[ref62] Kossek E. E. Roberts K. Fisher S. DeMarr B. (1998). Career self-management: a quasi-experimental assessment of the effects of a training intervention. Pers. Psychol. 51, 935–962. doi: 10.1111/j.1744-6570.1998.tb00746.x

[ref63] Kreiner G. E. Ashforth B. E. Sluss D. M. (2006). Identity dynamics in occupational dirty work: integrating social identity and system justification perspectives. Organ. Sci. 17, 619–636. doi: 10.1287/orsc.1060.0208

[ref64] Kreiner G. Mihelcic C. A. Mikolon S. (2022). Stigmatized work and stigmatized workers. Annu. Rev. Organ. Psychol. Organ. Behav. 9, 95–120. doi: 10.1146/annurev-orgpsych-012420-091423

[ref65] Lazarus R. S. (2006). Stress and Emotion: A New Synthesis. New York: Springer Publishing Company.

[ref66] Lazarus R. S. Folkman S. (1984). Stress, Appraisal, and Coping. New York: Springer Publishing Company.

[ref67] Le S. T. Lin S. P. (2023). Proactive personality and the job search outcomes: the mediating role of networking behaviour. Br. J. Guid. Couns. 51, 29–45. doi: 10.1080/03069885.2021.1998362

[ref68] Lee J. Y. Chen C. L. Kolokowsky E. Hong S. Siegel J. T. Donaldson S. I. (2021). Development and validation of the career crafting assessment (CCA). J. Career Assess. 29, 717–736. doi: 10.1177/10690727211002565

[ref69] Lent R. W. Brown S. D. (2013). Social cognitive model of career self-management: toward a unifying view of adaptive career behavior across the life span. J. Couns. Psychol. 60, 557–568. doi: 10.1037/a0033446, 23815631

[ref70] Lent R. W. Brown S. D. Hackett G. (1994). Toward a unifying social cognitive theory of career and academic interest, choice, and performance. J. Vocat. Behav. 45, 79–122. doi: 10.1006/jvbe.1994.1027

[ref71] Lent R. W. Ezeofor I. Morrison M. A. Penn L. T. Ireland G. W. (2016). Applying the social cognitive model of career self-management to career exploration and decision-making. J. Vocat. Behav. 93, 47–57. doi: 10.1016/j.jvb.2015.12.007

[ref72] Lent R. W. Ireland G. W. Penn L. T. Morris T. R. Sappington R. (2017). Sources of self-efficacy and outcome expectations for career exploration and decision-making: a test of the social cognitive model of career self-management. J. Vocat. Behav. 99, 107–117. doi: 10.1016/j.jvb.2017.01.002

[ref73] Lien S. S. Kosik R. O. Fan A. P. Huang L. Zhao X. Chang X. . (2016). 10-year trends in the production and attrition of Chinese medical graduates: an analysis of nationwide data. Lancet 388:S11. doi: 10.1016/S0140-6736(16)31938-9

[ref74] Lin N. (2002). Social Capital: A Theory of Social Structure and Action. Cambridge: Cambridge University Press.

[ref75] Lin S. H. Lu W. C. Chen M. Y. Chen L. H. (2014). Association between proactive personality and academic self-efficacy. Curr. Psychol. 33, 600–609. doi: 10.1007/s12144-014-9231-8

[ref76] Link B. G. Phelan J. C. (2001). Conceptualizing stigma. Annu. Rev. Sociol. 27, 363–385. doi: 10.1146/annurev.soc.27.1.363

[ref77] Little T. D. Cunningham W. A. Shahar G. Widaman K. F. (2002). To parcel or not to parcel: exploring the question, weighing the merits. Struct. Equ. Modeling 9, 151–173. doi: 10.1207/S15328007SEM0902_1

[ref78] Long J. S. Ervin L. H. (2000). Using heteroscedasticity consistent standard errors in the linear regression model. Am. Stat. 54, 217–224. doi: 10.1080/00031305.2000.10474549

[ref79] Marsh H. W. Wen Z. Hau K. T. (2004). Structural equation models of latent interactions: evaluation of alternative estimation strategies and indicator construction. Psychol. Methods 9, 275–300. doi: 10.1037/1082-989X.9.3.275, 15355150

[ref80] McAllister D. J. Kamdar D. Morrison E. W. Turban D. B. (2007). Disentangling role perceptions: how perceived role breadth, discretion, instrumentality, and efficacy relate to helping and taking charge. J. Appl. Psychol. 92, 1200–1211. doi: 10.1037/0021-9010.92.5.1200, 17845080

[ref81] Meara H. (1974). Honor in dirty work: the case of American meat cutters and Turkish butchers. Sociol. Work Occup. 1, 259–283. doi: 10.1177/073088847400100301

[ref82] Miller C. T. Kaiser C. R. (2001). A theoretical perspective on coping with stigma. J. Soc. Issues 57, 73–92. doi: 10.1111/0022-4537.00202

[ref83] Morales J. Lambert C. (2013). Dirty work and the construction of identity. An ethnographic study of management accounting practices. Account. Organ. Soc. 38, 228–244. doi: 10.1016/j.aos.2013.04.001

[ref84] Nalis I. Kubicek B. Korunka C. (2022). Resources to respond: a career construction theory perspective on demands, adaptability, and career crafting. Career Dev. Q. 70, 138–152. doi: 10.1002/cdq.12293

[ref85] National Health Commission of the People's Republic of China (2023). China Health Statistics Yearbook. Beijing: Peking Union Medical College Press.

[ref86] National Health Commission of the People's Republic of China (2024). Statistical Bulletin on the Development of China's Health Sector in 2023. Beijing: National Health Commission.

[ref87] Ni X. Zhu X. Bian W. Li J. Pan C. (2024). How does leader career calling stimulate employee career growth? The role of career crafting and supervisor-subordinate guanxi. Leadersh. Organ. Dev. J. 45, 21–34. doi: 10.1108/LODJ-07-2023-0400

[ref88] Noordegraaf M. (2007). From "pure" to "hybrid" professionalism: present-day professionalism in ambiguous public domains. Adm. Soc. 39, 761–785. doi: 10.1177/0095399707304434

[ref89] Noordegraaf M. (2011). Risky business: how professionals and professional fields (must) deal with organizational issues. Organ. Stud. 32, 1349–1371. doi: 10.1177/0170840611416748

[ref90] Park I. J. (2015). The role of affect spin in the relationships between proactive personality, career indecision, and career maturity. Front. Psychol. 6:1754. doi: 10.3389/fpsyg.2015.01754, 26635665 PMC4649026

[ref91] Parker S. K. Bindl U. K. Strauss K. (2010). Making things happen: a model of proactive motivation. J. Manage. 36, 827–856. doi: 10.1177/0149206310363732

[ref92] Parker S. K. Liao J. (2016). Wise proactivity: how to be proactive and wise in building your career. Organ. Dyn. 45, 217–227. doi: 10.1016/j.orgdyn.2016.07.007

[ref93] Parker S. K. Morgeson F. P. Johns G. (2017). One hundred years of work design research: looking back and looking forward. J. Appl. Psychol. 102, 403–420. doi: 10.1037/apl0000106, 28182465

[ref94] Parker S. K. Ohly S. (2008). “Designing motivating jobs: an expanded framework for linking work characteristics and motivation,” in Work Motivation: Past, Present, and Future, eds. Kanfer R. Chen G. Pritchard R. D. (New York: Routledge), 233–284.

[ref95] Parker S. K. Sprigg C. A. (1999). Minimizing strain and maximizing learning: the role of job demands, job control, and proactive personality. J. Appl. Psychol. 84, 925–939. doi: 10.1037/0021-9010.84.6.925, 10639910

[ref96] Parker S. K. Williams H. M. Turner N. (2006). Modeling the antecedents of proactive behavior at work. J. Appl. Psychol. 91, 636–652. doi: 10.1037/0021-9010.91.3.636, 16737360

[ref97] Petriglieri J. L. (2011). Under threat: responses to and the consequences of threats to individuals' identities. Acad. Manag. Rev. 36, 641–662. doi: 10.5465/amr.2009.0087

[ref98] Podsakoff P. M. MacKenzie S. B. Lee J. Y. Podsakoff N. P. (2003). Common method biases in behavioral research: a critical review of the literature and recommended remedies. J. Appl. Psychol. 88, 879–903. doi: 10.1037/0021-9010.88.5.879, 14516251

[ref99] Podsakoff P. M. MacKenzie S. B. Podsakoff N. P. (2012). Sources of method bias in social science research and recommendations on how to control it. Annu. Rev. Psychol. 63, 539–569. doi: 10.1146/annurev-psych-120710-100452, 21838546

[ref100] Preacher K. J. Hayes A. F. (2008). Asymptotic and resampling strategies for assessing and comparing indirect effects in multiple mediator models. Behav. Res. Methods 40, 879–891. doi: 10.3758/BRM.40.3.879, 18697684

[ref101] Rogers M. E. Creed P. A. (2011). A longitudinal examination of adolescent career planning and exploration using a social cognitive career theory framework. J. Adolesc. 34, 163–172. doi: 10.1016/j.adolescence.2009.12.010, 20106516

[ref102] Saks A. M. Zikic J. Koen J. (2015). Job search self-efficacy: reconceptualizing the construct and its measurement. J. Vocat. Behav. 86, 104–114. doi: 10.1016/j.jvb.2014.11.007

[ref103] Savickas M. L. (2013). “Career construction theory and practice,” in Career Development and Counseling: Putting Theory and Research to Work, eds. Brown S. D. Lent R. W.. 2nd ed (Hoboken: John Wiley & Sons), 144–180.

[ref104] Segars A. H. (1997). Assessing the unidimensionality of measurement: a paradigm and illustration within the context of information systems research. Omega 25, 107–121. doi: 10.1016/S0305-0483(96)00051-5

[ref105] Seibert S. E. Crant J. M. Kraimer M. L. (1999). Proactive personality and career success. J. Appl. Psychol. 84, 416–427. doi: 10.1037/0021-9010.84.3.416, 10380421

[ref106] Shantz A. Booth J. E. (2014). Service employees and self-verification: the roles of occupational stigma consciousness and core self-evaluations. Hum. Relat. 67, 1439–1465. doi: 10.1177/0018726713519280

[ref107] Slutskaya N. Simpson R. Hughes J. Simpson A. Uygur S. (2016). Masculinity and class in the context of dirty work. Gend. Work. Organ. 23, 165–182. doi: 10.1111/gwao.12119

[ref108] Spitzmuller M. Sin H. P. Howe M. Fatimah S. (2015). Investigating the uniqueness and usefulness of proactive personality in organizational research: a meta-analytic review. Hum. Perform. 28, 351–379. doi: 10.1080/08959285.2015.1021041

[ref109] Sturges J. Guest D. Conway N. Davey K. M. (2002). A longitudinal study of the relationship between career management and organizational commitment among graduates in the first ten years at work. J. Organ. Behav. 23, 731–748. doi: 10.1002/job.164

[ref110] Sun T. Wang J. Zhang S. Shi Y. Liu B. Wang X. (2021). Status, causes and consequences of physicians' self-perceived professional reputation damage in China: a cross-sectional survey. BMC Health Serv. Res. 21:344. doi: 10.1186/s12913-021-06306-6, 33853589 PMC8048359

[ref112] Tims M. Akkermans J. (2020). “Job and career crafting to fulfill individual career pathways,” in Career Pathways: From School to Retirement, eds. Hedge J. W. Carter G. W. (New York: Oxford University Press), 165–190.

[ref113] Tims M. Bakker A. B. Derks D. (2014). Daily job crafting and the self-efficacy—performance relationship. J. Manag. Psychol. 29, 490–507. doi: 10.1108/JMP-05-2012-0148

[ref114] Toyoki S. Brown A. D. (2013). Stigma, identity and power: managing stigmatized identities through discourse. Hum. Relat. 67, 715–737. doi: 10.1177/0018726713503024, 32842841

[ref115] van Leeuwen E. H. (2023). Contrasting views on the careers of classic professionals: exploring the careers of physicians. Eur. Manag. Rev. 20, 210–226. doi: 10.1111/emre.12531

[ref116] van Leeuwen E. H. Taris T. W. van den Heuvel M. Knies E. van Rensen E. L. J. Lammers J. W. J. (2021). A career crafting training program: results of an intervention study. Front. Psychol. 12:664453. doi: 10.3389/fpsyg.2021.664453, 34122255 PMC8187622

[ref117] van Leeuwen E. H. van den Heuvel M. Knies E. Taris T. W. (2020). Career crafting training intervention for physicians: protocol for a randomized controlled trial. JMIR Res Protoc. 9:e18432. doi: 10.2196/18432, 33030151 PMC7582143

[ref118] Vermooten N. Boonzaier B. Kidd M. (2019). Job crafting, proactive personality and meaningful work: implications for employee engagement and turnover intention. S. Afr. J. Ind. Psychol. 45, 1–13. doi: 10.4102/sajip.v45i0.1567

[ref119] Wang M. S. Raynard M. Greenwood R. (2020). From grace to violence: stigmatizing the medical profession in China. Acad. Manag. J. 64, 1842–1872. doi: 10.5465/amj.2018.0715

[ref111] Wass V. (2006). Doctors in society: medical professionalism in a changing world. Clin. Med. 6, 109–113. doi: 10.7861/clinmedicine.6-1-109 PMC4954417 16521367

[ref120] Westein M. P. D. de Vries H. Floor A. Koster A. S. Buurma H. (2019). Development of a postgraduate community pharmacist specialization program using CanMEDS competencies, and entrustable professional activities. Am. J. Pharm. Educ. 83:6863. doi: 10.5688/ajpe6863, 31507284 PMC6718509

[ref121] Wu H. Liu Y. (2022). The relationship between organisational support for career development, organisational commitment, and turnover intentions among healthcare workers in township hospitals of Henan, China. BMC Prim Care. 23:136. doi: 10.1186/s12875-022-01753-4, 35655133 PMC9161467

[ref122] Yang C. Luo Z. Xu N. Tang C. (2024). Fostering career crafting by developmental HR practices: the mediating role of future work self and moderating role of AI awareness. Career Dev. Int. 29, 641–655. doi: 10.1108/CDI-08-2023-0303

[ref123] Yildiz D. Bozbura F. T. Tatoglu E. Zaim S. (2024). How do organizational career management activities influence employees' career outcomes? The mediating role of career capital. Int. J. Organ. Anal. 32, 1805–1832. doi: 10.1108/IJOA-06-2023-3817

