# Supplementary material for: Proactive personality and career crafting in Chinese medical professionals: a conditional process model of occupational stigma and organizational career management
Source: Front Psychol. 2026 Aug 11;17:1876629. doi: 10.3389/fpsyg.2026.1876629 (PMC13503164; doi:10.3389/fpsyg.2026.1876629)
Supplement: Supplementary file 1 [file Table_1.DOCX]

**Supplementary Materials**

**1.Demographic information of participants**

| **TABLE S1 \|** Demographic information of participants | | | |
| --- | --- | --- | --- |
|  |  | ***N*** | Proportion (%) |
| Gender | Male | 65 | 21.104 |
|  | Female | 243 | 78.896 |
| Age | >18, <=20 | 10 | 3.247 |
|  | >20, <=25 | 55 | 17.857 |
|  | >25, <=30 | 101 | 32.792 |
|  | >30, <=35 | 45 | 14.610 |
|  | >35, <=40 | 29 | 9.416 |
|  | >40, <=45 | 18 | 5.844 |
|  | >45, <=50 | 38 | 12.338 |
|  | >50, <=64 | 12 | 3.896 |
| Education level | Junior College and below | 140 | 45.455 |
|  | Bachelor's degree | 159 | 51.623 |
|  | Master's degree | 5 | 1.623 |
|  | Doctoral degree | 4 | 1.299 |
| Type of job | Physician | 86 | 27.922 |
|  | Nurse | 103 | 33.442 |
|  | Researchers | 3 | 0.974 |
|  | Other professional and technical personnel | 58 | 18.831 |
|  | Logistics service personnel | 23 | 7.468 |
|  | Administrative personnel | 19 | 6.169 |
|  | Others | 16 | 5.195 |
| Title level | Junior | 220 | 71.429 |
|  | Intermediate | 61 | 19.805 |
|  | Associate senior | 23 | 7.467 |
|  | Senior | 4 | 1.299 |
| Job hierarchy | General staff | 215 | 69.805 |
|  | Junior management | 23 | 7.468 |
|  | Middle management | 56 | 18.182 |
|  | Senior management | 14 | 4.545 |
| Occupational tenure | <=1 | 27 | 8.766 |
|  | >1, <=3 | 44 | 14.286 |
|  | >3, <=5 | 33 | 10.714 |
|  | >5, <=10 | 88 | 28.571 |
|  | >10, <=20 | 63 | 20.455 |
|  | >20 | 53 | 17.208 |
| Organizational tenure | <=1 | 63 | 20.454 |
|  | >1, <=3 | 74 | 24.026 |
|  | >3, <=5 | 40 | 12.987 |
|  | >5, <=10 | 74 | 24.026 |
|  | >10, <=20 | 38 | 12.338 |
|  | >20 | 19 | 6.169 |
| Nature of hospital | Public | 160 | 51.948 |
|  | Private | 148 | 48.052 |
| Hospital level | Level 3A | 13 | 4.221 |
|  | Level 3B | 8 | 2.597 |
|  | Level 2A | 18 | 5.844 |
|  | Level 2B | 18 | 5.844 |
|  | Level 1A | 179 | 58.117 |
|  | Level 1B | 72 | 23.377 |

Note. N = 308. All nominal categorical variables were dummy-coded for regression analyses, with "Male", "Physician", "Public", and "Level 3A" as the reference groups. Ordinal categorical variables (age, education level, title level, job hierarchy, occupational tenure, organizational tenure) were treated as continuous.

**2.Confirmatory factor analysis results.**

**TABLE S2 |** Confirmatory factor analysis results.

| Model | *χ2/df* | RMSEA | SRMR | CFI | IFI | TLI |
| --- | --- | --- | --- | --- | --- | --- |
| 1. Six-factor（PP, CEDSE, CC, OS, OCM, CMV) | 2.098 | 0.060 | 0.063 | 0.913 | 0.914 | 0.908 |
| 2. Five-factor（PP, CEDSE, CC, OS, OCM) | 2.164 | 0.062 | 0.056 | 0.908 | 0.909 | 0.902 |
| 3. Four-factor（PP, CEDSE, CC, OS+OCM） | 2.703 | 0.074 | 0.080 | 0.865 | 0.866 | 0.857 |
| 4. Three-factor（PP+CEDSE, CC, OS+OCM） | 3.511 | 0.090 | 0.131 | 0.801 | 0.802 | 0.789 |
| 5. Two-factor（PP+CEDSE+OS+OCM, CC） | 4.563 | 0.108 | 0.155 | 0.717 | 0.718 | 0.700 |
| 6. One-factor（PP+CEDSE+OS+OCM+CC） | 5.177 | 0.117 | 0.174 | 0.668 | 0.669 | 0.648 |

Note. CMV = Common method variance. Model 1 includes an unmeasured latent method factor to assess common method bias. CC = Career Crafting, PP = Proactive Personality, CEDSE = Career Exploration and Decision-Making Self-Efficacy, OS = Occupational Stigma, OCM = organizational career management.

**3.Factor Loadings, Reliability, and Validity Metrics**

**TABLE s3|** Factor Loadings, Reliability, and Validity Metrics

|  |  | Factor Loadings | Item reliabilities | CR | AVE |
| --- | --- | --- | --- | --- | --- |
| **Proactive personality** | | | | 0.911 | 0.509 |
|  | Item1 | 0.649 | 0.421 |  |  |
|  | Item2 | 0.683 | 0.466 |  |  |
|  | Item3 | 0.616 | 0.379 |  |  |
|  | Item4 | 0.675 | 0.456 |  |  |
|  | Item5 | 0.689 | 0.475 |  |  |
|  | Item6 | 0.689 | 0.475 |  |  |
|  | Item7 | 0.776 | 0.602 |  |  |
|  | Item8 | 0.728 | 0.530 |  |  |
|  | Item9 | 0.808 | 0.653 |  |  |
|  | Item10 | 0.793 | 0.629 |  |  |
| **Career crafting** | | | | 0.974 | 0.716 |
| changing relational boundaries | Item1 | 0.906 | 0.821 |  |  |
|  | Item2 | 0.896 | 0.803 |  |  |
|  | Item3 | 0.876 | 0.767 |  |  |
|  | Item4 | 0.908 | 0.824 |  |  |
| utilizing relational resources | Item5 | 0.880 | 0.774 |  |  |
|  | Item6 | 0.746 | 0.557 |  |  |
|  | Item7 | 0.734 | 0.539 |  |  |
|  | Item8 | 0.840 | 0.706 |  |  |
| reflecting positive career meaning | Item9 | 0.851 | 0.724 |  |  |
|  | Item10 | 0.794 | 0.630 |  |  |
|  | Item11 | 0.827 | 0.684 |  |  |
|  | Item12 | 0.841 | 0.707 |  |  |
| expanding task boundaries | Item13 | 0.772 | 0.596 |  |  |
|  | Item14 | 0.918 | 0.843 |  |  |
|  | Item15 | 0.875 | 0.766 |  |  |
| **Career exploration and decision-making self-efficacy** | | | | 0.973 | 0.748 |
|  | Item1 | 0.890 | 0.792 |  |  |
|  | Item2 | 0.909 | 0.826 |  |  |
|  | Item3 | 0.905 | 0.819 |  |  |
|  | Item4 | 0.847 | 0.717 |  |  |
|  | Item5 | 0.932 | 0.869 |  |  |
|  | Item6 | 0.936 | 0.876 |  |  |
|  | Item7 | 0.906 | 0.821 |  |  |
|  | Item8 | 0.930 | 0.865 |  |  |
|  | Item9 | 0.733 | 0.537 |  |  |
|  | Item10 | 0.784 | 0.615 |  |  |
|  | Item11 | 0.796 | 0.634 |  |  |
|  | Item12 | 0.776 | 0.602 |  |  |
| **Organizational career management** | | | | 0.942 | 0.619 |
|  | Item1 | 0.711 | 0.506 |  |  |
|  | Item2 | 0.797 | 0.635 |  |  |
|  | Item3 | 0.857 | 0.734 |  |  |
|  | Item4 | 0.795 | 0.632 |  |  |
|  | Item5 | 0.786 | 0.618 |  |  |
|  | Item6 | 0.809 | 0.654 |  |  |
|  | Item7 | 0.632 | 0.399 |  |  |
|  | Item8 | 0.823 | 0.677 |  |  |
|  | Item9 | 0.786 | 0.618 |  |  |
|  | Item10 | 0.844 | 0.712 |  |  |
| **Occupational stigma** | | | | 0.879 | 0.551 |
|  | Item1 | 0.605 | 0.366 |  |  |
|  | Item2 | 0.674 | 0.454 |  |  |
|  | Item3 | 0.787 | 0.619 |  |  |
|  | Item4 | 0.780 | 0.608 |  |  |
|  | Item5 | 0.877 | 0.769 |  |  |
|  | Item6 | 0.697 | 0.486 |  |  |

**4.Measurement Scales: Original English Items and Chinese Translations**

| Construct / Scale Name | | Original English Item | Final Chinese Translation | Notes on Contextual Adaptation |
| --- | --- | --- | --- | --- |
| Proactive Personality (Seibert et al, 1999) | | 1. I am constantly on the lookout for new ways to improve my life. | 1.我一直在寻找新方法来改善我的生活。 | No adaptation needed. |
|  |  | 2. Wherever I have been, I have been a powerful force for constructive change. | 2.无论我走到哪里，我都是推动建设性变革的强大力量。 | No adaptation needed. |
|  |  | 3. Nothing is more exciting than seeing my ideas turn into reality. | 3.没有什么比看到我的想法变成现实更令人兴奋的了。 | No adaptation needed. |
|  |  | 4. If I see something I don't like, I fix it. | 4.如果我看到我不喜欢的东西，我就纠正它。 | No adaptation needed. |
|  |  | 5. No matter what the odds, if I believe in something I will make it happen. | 5.不管概率有多大，只要我相信某件事，我就会让它发生。 | No adaptation needed. |
|  |  | 6. I love being a champion for my ideas, even against others' opposition. | 6.我喜欢成为自己想法的捍卫者，即便自己的想法遭到了别人的反对。 | No adaptation needed. |
|  |  | 7. I excel at identifying opportunities. | 7.我擅长发现机会。 | No adaptation needed. |
|  |  | 8. I am always looking for better ways to do things | 8.我一直在寻找更好的做事方法。 | No adaptation needed. |
|  |  | 9. If I believe in an idea, no obstacle will prevent me from making it happen. | 9.如果我相信一个想法，没有任何障碍可以阻止我实现它。 | No adaptation needed. |
|  |  | 10. I can spot a good opportunity long before others can. | 10.我能比别人更早地发现好机会。 | No adaptation needed. |
| Career Exploration and Decision Self-Efficacy  (Lent, et al., 2016) | Decisional Coping Efficacy | 1. figure out which career options could provide a good fit for your personality | 1. 找到适合我的性格的职业。 | No adaptation needed. |
|  |  | 2. identify careers that best use your skills. | 2. 找到最能发挥我技能的职业。 | No adaptation needed. |
|  |  | 3. pick the best-fitting career option for you from a list of your ideal careers | 3. 从我的理想职业列表中选择出最适合我的职业。 | No adaptation needed. |
|  |  | 4. learn more about careers you might enjoy | 4. 了解更多我可能喜欢的职业的信息。 | No adaptation needed. |
|  |  | 5. match your skills, values, and interests to relevant occupations | 5. 将我的技能、价值观和兴趣与相关的职业相匹配。 | No adaptation needed. |
|  |  | 6. make a well-informed choice about which career path to pursue | 6. 在对职业进行充分了解的情况下选择我的职业道路。 | No adaptation needed. |
|  |  | 7. learn more about jobs that could offer things that are important to you | 7. 多了解对我来说重要的工作。 | No adaptation needed. |
|  |  | 8. identify careers that best match your interests | 8. 确定最符合我兴趣的职业。 | No adaptation needed. |
|  | Decisional Coping Efficacy | 1. cope with the disappointment if your first choice does not work out | 1. 如果第一个职业选择不成功，我能应对失望。 | No adaptation needed. |
|  |  | 2. manage negative feelings if your first choice does not work out and you need to find another career choice | 2. 如果第一个职业选择行不通，我能管理好负面情绪，找到第二个职业选择。 | No adaptation needed. |
|  |  | 3. create a good back-up plan if you discover that you don't have enough financial support to pursue your preferred career option | 3. 如果我发现自己没有足够的经济支持来追求自己喜欢的职业，我能制定一个好的后备计划。 | No adaptation needed. |
|  |  | 4. develop a good alternative plan if you find that your access to your most preferred option is blocked for some reason | 4. 如果我发现最喜欢的职业选择因为某种原因被阻止了，我能制定一个好的替代计划。 | No adaptation needed. |
| Occupational Stigma  (Shantz and Booth, 2014) | | 1. Most people who are not call center workers have a lot more negative thoughts about call center workers than they actually express | 1.他们对我们医疗人员有很多负面的想法，即使他们没有表达出来。 | “call center employees” replaced by “medical professionals” |
|  |  | 2. Most people who are not call center workers have a problem viewing call center workers as equals | 2.非医疗人员很难以平常的眼光看待我们。 | “call center employees” replaced by “medical professionals |
|  |  | 3. Most people who are not call center workers judge call center workers on the basis of their job and the work that they do on the job | 3.非医疗人员会根据我从事的职业和工作来对我进行判断。 | “call center employees” replaced by “medical professionals” |
|  |  | 4. My being a call center worker influences how people who are not call center workers act with me | 4.我所从事的职业影响了他们对我的态度。 | “Call center employees” replaced by “medical professionals |
|  |  | 5. When interacting with people who are not call center workers, I feel like they interpret all my behaviors in terms of the fact that I am a call center worker | 5.当我与他们互动时，他们会把我所有的行为归因为我的职业。 | “Call center employees” replaced by “medical professionals |
|  |  | 6. Stereotypes about call center workers have affected me personally | 6.对医疗人员的刻板印象对的我个人生活产生了影响。 | “Call center employees” replaced by “medical professionals |
| Organizational Career Management Support  (Sturges et al., 2002 ) | | 1. I have been given training to help develop my career | 1. 组织经常提供有助于我职业发展的培训。 | No adaptation needed. |
|  |  | 2. My boss has made sure I get the training I need for my career | 2. 我的领导或上司确保我能得到职业发展所需的培训。 | No adaptation needed. |
|  |  | 3. I have been taught things I need to know to get on in this organization | 3. 我学习了在这个组织中生存需要知道的事情。 | No adaptation needed. |
|  |  | 4. I have been given a personal development plan | 4. 组织为我制定了一个个人发展计划。 | No adaptation needed. |
|  |  | 5. I have been given work which has developed my skills for the future | 5. 我得到了一份能培养我未来技能的工作。 | No adaptation needed. |
|  |  | 6. My boss has give me clear feedback on my performance | 6. 我的领导或上司对我的表现给予了明确的反馈。 | No adaptation needed. |
|  |  | 7. I have been given impartial career advice when I needed it | 7. 在我需要的时候，我得到了公正的职业建议。 | No adaptation needed. |
|  |  | 8. I have been introduced to people at work who are prepared to help me develop my career | 8. 我被介绍给一些愿意帮助我职业发展的同事。 | No adaptation needed. |
|  |  | 9. I have been given a mentor to help my career development | 9. 我有一位帮助我职业发展的导师。 | No adaptation needed. |
|  |  | 10. My boss has introduced me to people who will help my career | 10. 我的领导或上司给我介绍了一些对我的职业有帮助的人。 | No adaptation needed. |
| Career Crafting  (Lee et al, 2021) | Changing Relational Boundaries | 1. I make an effort to get to know people whose career I admire | 1. 我努力去了解我所钦佩的人。 | No adaptation needed. |
|  |  | 2. I make connections with people who share my career Interests | 2. 我与那些和我有共同职业兴趣的人建立联系。 | No adaptation needed. |
|  |  | 3. I make connections with people who have the skills I want to develop in my career | 3. 我和那些拥有我追求的职业发展相关技能的人建立联系。 | No adaptation needed. |
|  |  | 4. I build contacts with people in areas where I would like to work. | 我与我喜欢的工作领域的人建立联系。 | No adaptation needed. |
|  | Utilizing Relational Resources | 1. I seek professional coaching from those whose careers I admire | 1. 我向我钦佩的人寻求专业指导。 | No adaptation needed. |
|  |  | 2. I attend events that will help me explore different career paths | 2. 我参加一些帮助我探索不同职业道路的活动。 | No adaptation needed. |
|  |  | 3. I ask others to introduce me to people who can positively influence my career. | 3. 我请别人把我介绍给那些能对我的事业产生积极影响的人。 | No adaptation needed. |
|  |  | 4. I ask others for feedback on my career development | 4. 我征求别人对我职业发展的反馈。 | No adaptation needed. |
|  | Reflecting Positive Career Meaning | 1. I think about the ways in which my career positively impacts my life. | 1. 我会思考给我的生活带来积极影响的职业路径和方式。 | No adaptation needed. |
|  |  | 2. I remind myself that my career has personal significance | 2. 我提醒自己，我的职业对我个人有重要意义。 | No adaptation needed. |
|  |  | 3. I reflect on the role my career has for my overall well-being | 3. 我反思我的职业生涯对我的整体幸福所起的作用。 | No adaptation needed. |
|  |  | 4. I look at a career as a means of expressing myself | 4. 我把职业生涯看作是表达自我的一种方式。 | No adaptation needed. |
|  | Expanding Task Boundaries | 1. I choose to take on additional tasks at work | 1. 我选择在工作中承担额外的任务。 | No adaptation needed. |
|  |  | 2. I take on extra tasks that contribute to my career even if I do not receive extra pay for them. | 2. 即使没有额外的报酬，我也会做一些对我的职业生涯有帮助的额外工作。 | No adaptation needed. |
|  |  | 3. Added work responsibilities excite me when they are relevant to my career interests. | 3. 当增加的工作职责与我的职业兴趣相关时，我会感到兴奋 | No adaptation needed. |
